# Trident Nano-Indexing the Proteomics Table: Next-Version Clustering of Iron Carbide NPs and Protein Corona

**DOI:** 10.3390/molecules27185754

**Published:** 2022-09-06

**Authors:** Murtaza Hasan, Ayesha Zafar, Maryum Jabbar, Tuba Tariq, Yasmeen Manzoor, Muhammad Mahmood Ahmed, Shahbaz Gul Hassan, Xugang Shu, Nasir Mahmood

**Affiliations:** 1 School of Chemistry and Chemical Engineering, Zhongkai University of Agriculture and Engineering, Guangzhou 510225, China; 2 Department of Biotechnology, The Islamia University of Bahawalpur, Bahawalpur 63100, Pakistan; 3 Department of Biomedical Engineering, College of Future Technology, Peking University, Beijing 100871, China; 4 College of Information Science and Engineering, Zhongkai University of Agriculture and Engineering, Guangzhou 510225, China; 5 School of Science, RMIT University, Victoria 3000, Australia

**Keywords:** nanoparticle corona, proteomics table, biomarker, signaling, biomaterials, early disease detection, cancer

## Abstract

Protein corona composition and precise physiological understanding of differentially expressed proteins are key for identifying disease biomarkers. In this report, we presented a distinctive quantitative proteomics table of molecular cell signaling differentially expressed proteins of corona that formed on iron carbide nanoparticles (NPs). High-performance liquid chromatography/electrospray ionization coupled with ion trap mass analyzer (HPLC/ESI-Orbitrap) and MASCOT helped quantify 142 differentially expressed proteins. Among these proteins, 104 proteins showed upregulated behavior and 38 proteins were downregulated with respect to the control, whereas 48, 32 and 24 proteins were upregulated and 8, 9 and 21 were downregulated CW (control with unmodified NPs), CY (control with modified NPs) and WY (modified and unmodified NPs), respectively. These proteins were further categorized on behalf of their regularity, locality, molecular functionality and molecular masses using gene ontology (GO). A STRING analysis was used to target the specific range of proteins involved in metabolic pathways and molecular processing in different kinds of binding functionalities, such as RNA, DNA, ATP, ADP, GTP, GDP and calcium ion bindings. Thus, this study will help develop efficient protocols for the identification of latent biomarkers in early disease detection using protein fingerprints.

## 1. Introduction

Scanning and recognition of molecular biomarkers with unique synthetic and physiological identities for limiting metabolic reactions of cells [1], tissue, or living organism exudates always remain difficult tasks in the field of nanomedicine for early detection [2] and treatment of diseases [3]. Proteome cataloguing of cancer cells and many other human disease cell fluids is potentially considered to be an important and rich source for biomarker discoveries [4], as these fluids contain a variety of enzymes which are proteins in nature [5]. These proteins control a wide range of functions within the living organism, such as metabolic reaction [6], cell signaling, transporting molecules from one location to another location and responding to stimuli such as metabolites, microgravity and NPs [7,8]. The most interesting and fascinating aspect of nanomaterials is the formation of a corona around the nanoparticles (NPs) [9]. The protein corona differs with NP size, shape, structure [10] and surface chemistry [11]. However, there are only a few reports regarding the exact understanding of the relationship of NPs with living cells or their exudates. 

The investigations of various proteins having unique functions in NP corona have become crucial in the field of nanomedicine for therapy and treatment. However, the complex NP–protein interactions made it difficult, for reasons such as peculiar biodistribution, unexpected toxicity and unwanted signals [12]. For example, a recent study found that introduction of NPs altered the physiological status of the vascular endothelial cadherin protein, with devastating endothelial cell leakiness [13]. In another report, researchers used modified nanomaterials which created complex protein corona and influenced active targeting, drug release fluctuations in cellular uptake and intracellular trafficking of NPs [14]. However, it is believed that a group of proteins such as opsonin have the ability to bind with NPs which control the phagocytosis process [15], while coating NPs with different surfactants enhances their efficiency [16]. Moreover, it is also known that variation in the composition of human plasma in each disease also altered the profiling of novel biomarkers from biological fluids [17]. In addition, even normal people also have variations in their plasma composition with respect to genetic variability and differences in age, gender and lifestyle [18]. Therefore, it is highly required to investigate the sensitive proteins [12] which are impacted by the NP and cell interactions, hence changing the biological signaling [19]. Moreover, the nano–bio interaction, and especially the personalized protein corona profiling relevant to disease and NPs, is compulsory for development within the field to accelerate more predictable results in detection, diagnosis and treatment at the clinical level [20]. Among various NPs, iron carbide NPs have obtained great attention as being abundant molecules with a higher compatibility factor. The recoded studies show iron carbide consists of optimized active phases, functions as the active phase for activation of process and provides thermodynamic stability and selectivity for specific functioning. In addition, the structure–performance relationship becomes enhanced by adopting a green synthesis route that displays a green chemistry over the entire molecule, as well as biocompatibility [21,22]. In this work, *Withania* extract and iron precursors were used that provided us with iron carbide NPs. Green synthesis is a cascade of reactions between the precursor and the biomolecules, where the biomolecules function as the reducing and capping agent for the synthesis of NPs, giving specific morphology and surface chemistry [23,24,25,26,27]. The entire green synthesis involves additional and replacement reactions that provide us with iron carbide NPs. Gold particles or any other expensive metal were unaffordable for us, therefore they could not be used. In addition, the structure complexity, stability and selectivity of iron carbide NPs provides stable configuration of protein corona. Such physiochemical and green chemistry of particles provided a more stable structure to hold proteins and later facilitate further studies. Thereby, we concluded that green iron carbides hold the proteins well and show higher compatibility. This trial introduces more applications of green synthesized iron carbide NPs. The analytically accessible layer of the protein/biomolecule describes the corona protein that is chiefly bound using noncovalent bonds such as hydrogen bonds, solvation forces and Van der Waals interactions [28], where low-affinity proteins bind first and then are replaced by ones with greater affinity [29,30]. In addition, high-affinity proteins present in low concentrations replace low-affinity proteins at high concentrations. This corona changes over time by a large interchange of proteins that governs the hard protein corona. Protein corona profiling accelerates the clinical translation of nanomedicines and facilitates the identification of disease-specific biomarkers. 

In this study, we introduced a novel method of separating unique protein fingerprints, and their differential expression in the form of a proteomics table from HeLa cells in response to carboxylate-coated Fe_2_C NPs. A unique proteomics table was designed with changing molecular masses, functionality, patterns of regulation and protein–protein interactions in columns and rows to provide a platform to give clues for early disease detection via proteomics studies. This study will be helpful for researchers to set a basic criterion for altering the protein expression, masses and molecular function in response to outer stimuli.

## 2. Results and Discussion

**Figure 1:**
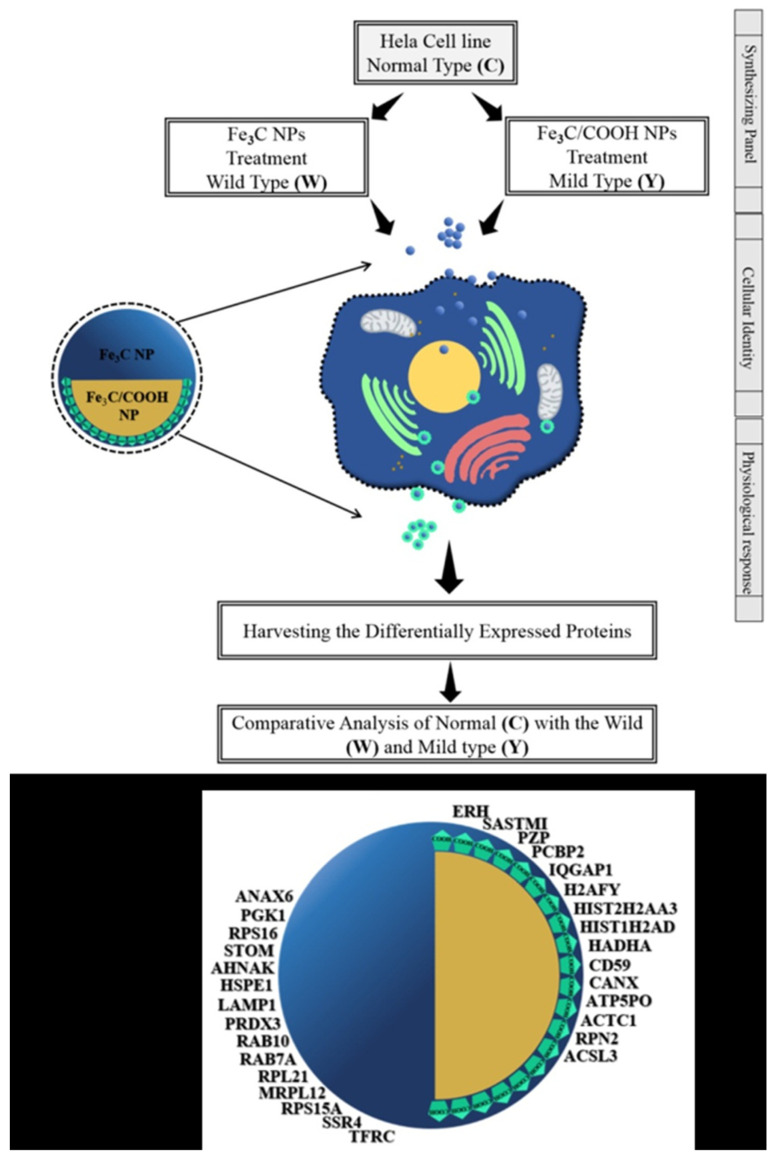
Schematic illustration representing the differentially expressed proteins from the protein corona on Fe_2_C NPs via nano–bio interaction from HeLa cells.

The differentially expressed proteins formed a unique protein corona which has critical information on cancer cells (HeLa cells) in response to NPs. HeLa cells are a cell line that can grow and divide endlessly in a laboratory, which has contributed to their adoption across the world as the human cell line of choice for biomedical research. Figure 1 presents the schematic illustration of differential expression of protein corona in response to unmodified and modified Fe_2_C NPs and their identification. The synthesizing panel focuses on a HeLa cell line represented as the control, which was treated with two distinct groups of synthesized NPs characterized as W or wild type (bare Fe_2_C NPs), and the Y as a mild type (Fe_2_C-COOH NPs). The difference in the surface chemistry enhances the effective entanglement of proteins and generates specific protein corona. Finally, differentially expressed proteins were harvested to investigate the cellular identity and physiological response of differentially expressed proteins in each case individually, collectively and comparatively. Three major groups of differentially identified proteins were classified as tamed (CW), mild (CY) and wild (YW) after a comparative analysis among C, W and Y. Moreover, the double targeting strategy re-categorized the harvested proteins into Nexus and distinct differentially expressed cancerous proteins, thus providing a remarkable set of cancer-inducing protein biomarkers using the double shooting technique. 

Before employing the as-synthesized NPs to cells, the biologically synthesized NPs were examined using UV-vis spectrometry and compared with chemically reduced NPs. Figure S1a clearly shows prominent peaks at 295 and 280 nm for chemical and bio-reduced NPs, respectively, confirming that bio-reduced NPs have similar properties to chemical ones. Furthermore, Fourier transform infrared spectroscopy (FTIR) was used to determine the surface chemistry of modified and bare NPs, and a peak at 1500 cm^−1^ confirmed the successful coating of NPs with the carboxyl group (Figure S1b). The peak at 3000 cm^−1^ represents the C-H binding, which assists the attachment of the carboxyl groups to NPs. 

**Figure 2:**
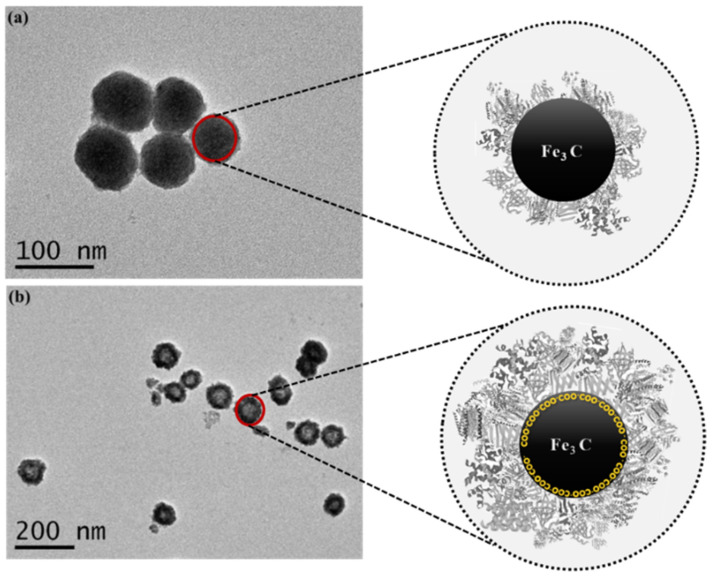
TEM images of (**a**) non-coated Fe_2_C NPs and (**b**) COOH coated Fe_2_C NPs, while zoomed images show possible protein corona.

Transmission electron microscopy (TEM) was used to confirm any change in the shape and/or size of NPs due to surface functionalization. It is found that the surface of coated NPs becomes rough with an increase in size by 30–50 nm (Figure 2). The increase in the size is due to modification, which resulted in hard protein corona. The functionalization increased the adhering capability due to the enhanced surface compatibility, hence providing ease in harvesting all deferentially expressed proteins [31]. 

### 2.1. Identification and Quantification of Differentially Expressed Proteins

The differentially expressed protein corona of Hela cells formed around the NPs, which were harvested and characterized by using LC/MS mass spectrometry. The 142 identified proteins were quantified by HPLC/MS/TOF and classified by the SWISS PROT protein database and MASCOT server, as listed in Table S1.

### 2.2. Differential Expression Based on Regulation

**Figure 3:**
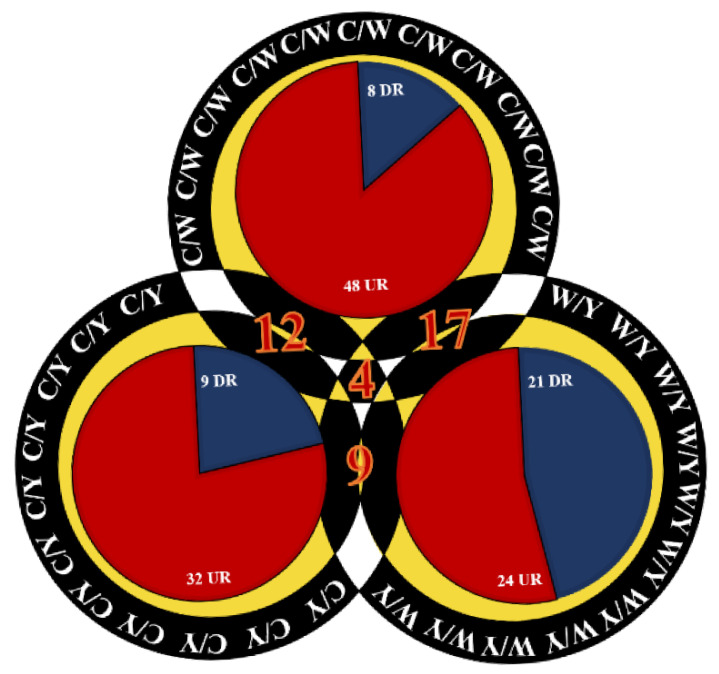
Venn diagram relationship of differentially expressed proteins on basis of regularity within the CW, CY and YW groups.

The differential expression of identified proteins was analyzed based on the regulation of these proteins in response to incubation of NPs [32]. To determine the significance value of differentially expressed proteins, a threshold value was set from 0.67–1.67. Proteins that had a threshold value below 0.67 were categorized as upregulated proteins and above 1.67 were classified as downregulated proteins. Out of a total of 142 identified proteins, 104 were upregulated and 38 were downregulated. Regularity was further ordered according to three major groups, i.e., CW, CY and YW. A clear relationship among tame (Table S2), mild (Table S1) and wild (Table S4) was found that could describe the common proteins which were repeated, overlapped and present in all three systems to display the significance of double targeting of coated and bare NPs. The comparison showed that tame–mild, tame–wild and mild–wild had 12, 17 and 9 proteins in common, respectively (Tables S5–S7). The proteins that were present in all of them are four in number, which may be interdependent and play a vital role in illuminating the cancerous proteins (Table S8). The further deep analysis highlighted a unique set of proteins that include the variant set of proteins in the tame (Table S9), mild (Table S10) and wild (Table S11) groups. These were marked as the unique proteins scavenged using the particles that enlarged the proteomic pools, providing blueprints of unique categories in the respective group, thereby displaying the common, unique and comparative proteins using a trident NP. In addition, the Venn diagram shows the regulation of differentially expressed proteins as upregulated: downregulated, where tame showed 48(86%):8(14%), mild showed 32 (78%):9(22%) and wild showed 24(53%):21(47%) conclusively, shown in Figure 3.

### 2.3. Proteomics Table

**Figure 4:**
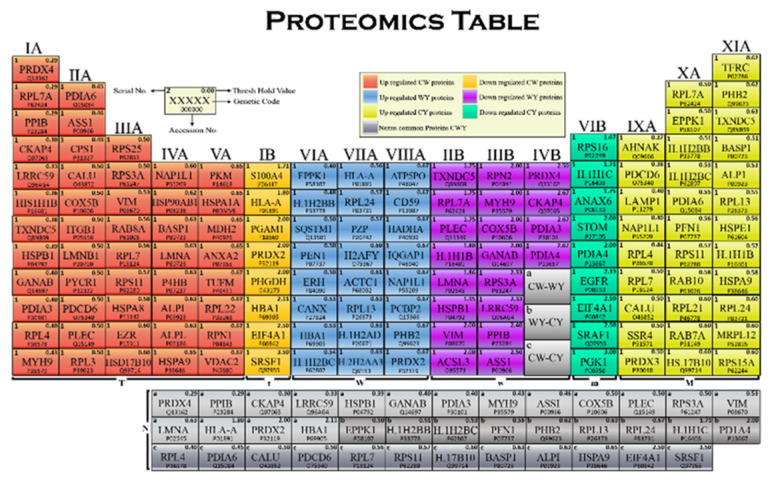
Proteomics table providing quantitative analysis of all differentially expressed proteins in CW, CY and YW groups.

The tabular arrangement of harvested differentially expressed proteins is represented in the proteomics table by the increasing threshold values of each protein in respective groups and in order of their regularity (Figure 4). Each cell of the table consists of the serial number from 1–142 proteins, protein genetic code, accession no. assigned to the protein and the threshold value of all differentially expressed proteins. The proteomics table consists of 16 vertical columns and 12 horizontal rows that are arranged for grouping the proteins in a distinguishable manner. The columns are arranged by the increasing threshold values of the respective protein per assigned block, whereas the rows are arranged to give simplified alignment.

The column of elements is called a group or family, arranged in order of their regularity and differentiation. The groups are numbered as I–XVI and sub-classified into A and B: “A” represents the upregulated group and “B” represents the downregulated group. The blocks are sections of a proteomics table that shows the classification on the bases of comparative and regulatory analysis. It consists of seven blocks: T, t, W, w, M, m and N. Group IA-VA is the T-block, consisting of the 48 tame upregulated proteins, while the t-block consists of the 8 tame downregulated proteins in group IB. Group VIA-VIIIA is the W-block, consisting of the 24 wild-type upregulated proteins, and the w-block consists of 21 downregulated wild-type proteins in group IIB-IVB. Next, the M-block comprises 32 mild upregulated proteins in group IXA-XIA, with m-block having 9 mild-type downregulated proteins in the VIB group. The N-block is the last block of the proteomics table, known as Nexus proteins that are classified as a, b and c, where a is the Nexus of the tame–mild group, containing 12 proteins, b is the Nexus of the tame–wild group, with 17 proteins, and c is the Nexus of the mild–wild group, bearing 9 proteins. 

**Figure 5:**
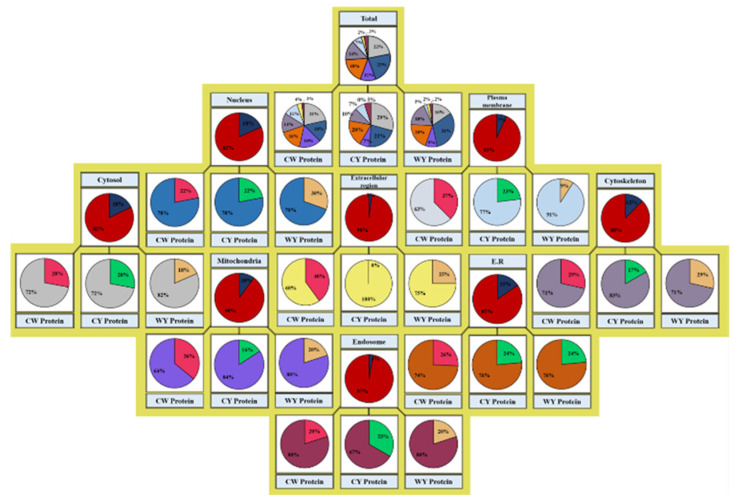
Classification of differentially expressed protein based on subcellular localization of identified proteins.

Further, subcellular localization of the identified proteins was determined by GO analysis and the uniport database, and the results are presented in the form of percentages in Figure 5. Out of 142 proteins, 22%, 23%, 11%, 18%, 14%, 7%, 2% and 3% were present in cytosol, mitochondria, ER, cytoskeleton, plasma membrane, extracellular region and in endosomes, respectively. Figure 5 also compares the percentages of identified proteins as tame: mild: wild (total 56:41:45) 21%:29%:16% in cytosol, 16%:22%:31% in nuclei, 16%:7%:9% in mitochondria, 16%:20%:20% in ER, 14%:10%:18% in cytoskeleton, 11%:7%:2% in plasma membrane, 4%:0%:2% in extracellular region and 2%:5%; 2% in endosomes, respectively. Furthermore, we also simplified the results on the basis of individual organelles following the same ratios, with 18% of the total identified proteins located in nuclei as 22%: 22%: 30%, 7% of plasma membrane as 37%:23%:9%, 18% of cytosol as 28%:28%:18%, 12% of cytoskeleton as 29%:17%:29%, 2% of extracellular region as 40%:0%:25%, 10% of mitochondria as 36%:16%:20%, 15% of E.R as 26%:24:24% and 3% of endosomes as 20%:33%:20%, respectively.

**Figure 6:**
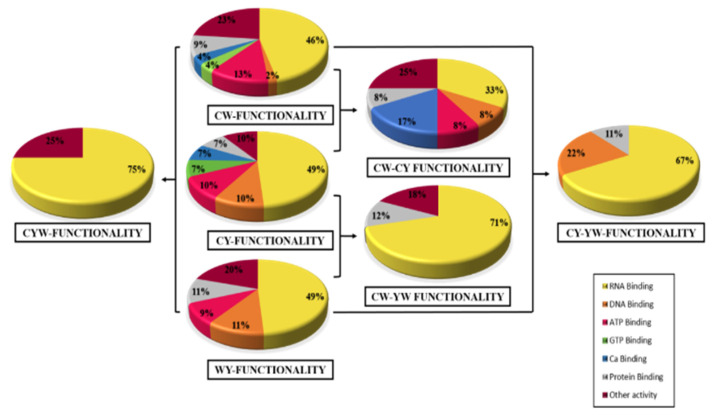
Classification of differentially expressed protein based on the functionalities of identified proteins.

To obtain further insights, a comparative analysis was made between the functionality of differentially expressed proteins in different groups, i.e., tame, mild and wild (56:41:45). The results showed that these groups proteins comprised 46%:49%:49% RNA, 2%:10%:11% DNA, 13%:10%:9% ATP, 4%:7%:0% GTP, 4%:7%:0% calcium ion, 9%:7%:11% other protein-binding proteins and 23%:10%:20% different activity proteins (Figure 6). In addition, 12 proteins were found to be common in tame–mild, having 33% RNA, 8% ATP/ADP, 8% DNA, 17% calcium ion, 8% protein-binding proteins and 25% different activity proteins. Similarly, tame–wild had nine proteins in common, out of which 71% (RNA) and 12% (protein) were binding and 18% other activity proteins. Mild–wild had 17 proteins in common, containing 67% RNA, 22% DNA and 11% different types of protein-binding proteins. However, there were four common proteins in tame–mild–wild, which comprised 75% RNA binding proteins and 25% other activity proteins. 

**Figure 7:**
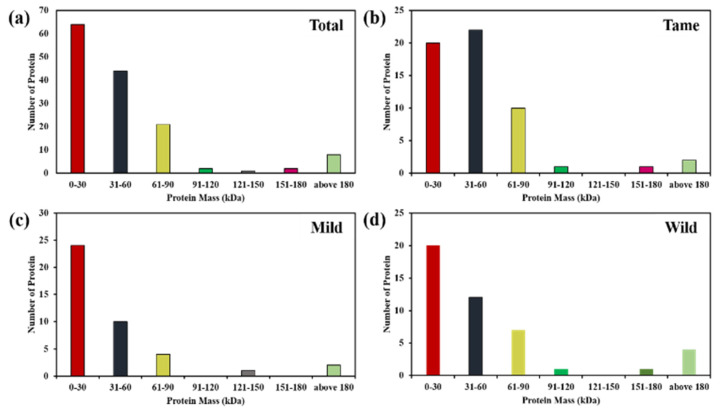
Comparative analysis of identified total protein CWY (**a**) Tame Protein CW (**b**), Mild Protein CY (**c**) and Wild Protein WY (**d**) on the basis of molecular masses.

Further comparison was made based on relative abundance of identified proteins. The proteins were quantified by different ranges of molecular masses (kDa) starting from 0–180 kDa, and evaluation was given in the form of bar charts (Figure 7). The comparative analysis of total identified protein in tame–mild–wild demonstrated that out of 142 differentially expressed proteins, 64, 44, 21, 2, 1, 2 and 8 proteins have molecular masses in the range of 0–30 kDa, 31–60 kDa, 61–90 kDa, 91–120 kDa, 121–150 kDa, 151–180 kDa and above 180 kDa, respectively. Based on the proteins’ masses, the respective protein numbers in individual tame: mild: wild are 20:24:20, 22:10:12, 10:4:7, 1:0:1, 0:1:0, 1:0:1 and 2:2:4, respectively.

**Figure 8:**
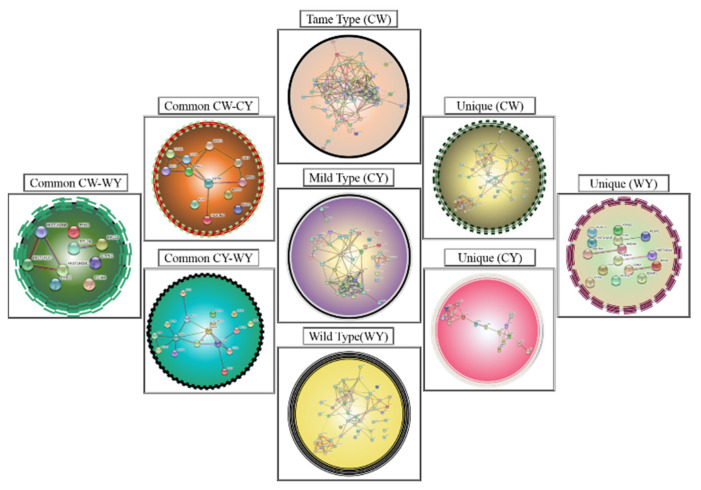
A STRING analysis showing the protein–protein interaction of differentially expressed proteins.

After identifying and classifying the proteins, a STRING algorithm (search tool for retrieval of interacting genes/protein) was used to create protein–protein interactions for a better understanding of the molecular mechanism and biological metabolism that shows cellular responses activated by the wild and mild type. The interaction showed the linkage between total identified proteins, called the interatomic of organisms, as shown in Figure 8. This interaction helped to evaluate the responsive action of different downregulated and upregulated proteins in the presence of NPs, as explained below.

The study was designed for cancer biomarkers where the NPs will effectively capture the differential proteins and provide the biomarkers using gene ontology and proteomics. On the other hand, if other cells are used, NPs will provide specific and selective ranges of proteins depending on the cell line. The NPs act as specific probes that effectively capture differentially expressed proteins, as the NPs bear higher stability and biocompatibility for proteins but the cell line and their protein expression will vary according to certain diseases and biomarkers, therefore the abundance and affinity of protein varies to according to the disease or defect.

Several proteins were downregulated in response to NPs, such as S100A4, which regulates the calcium-binding protein and is involved in the regulation of several cellular processes, such as cell cycle progression and differentiation [33]. Confirmation of this protein from previously reported studies show that it controls the function in motility, invasion and tubulin polymerization, and altered expression of this gene has been implicated in tumor metastasis [34,35]. The onset of metastasis happens after suppressed cell growth, migration and invasion activities when it is downregulated. As the studies show that downregulation of S100A4 mediates suppression of cell growth in colorectal cancer, metastasis reduces the chance of breast cancer and decreases cell proliferation and invasion in thyroid cancer. Another downregulated protein, phosphoglycerate mutase 1, is an important glycolytic enzyme that catalyzes the conversion of 3-phosphoglycerate to 2-phosphoglycerate, which was downregulated in presence of Fe_2_CNPs. Its main function is to catalyze the reaction and transfer the phosphate group from one carbon compound to another, which controls the ATP synthesis, hence interaction with NPs induced the cell to produce more ATP [34,35]. The downregulation confers clinical prognostic significance by inhibiting the proliferation of colorectal cancer cells [36,37]. Normally, this protein controls the transfer of electrons under oxidative stress and alters the serine pathway flux, which might increase the chances of other carcinomas. Our results also confirmed that upregulation of this protein induces other carcinoma types, as shown in Table S5. This is a result of lower plasma serine levels, loss of DNA methylation, mutational changes, mitochondrial dysfunction and transcriptional downregulation for provoking oncogenic pathways [38,39]. However, the downregulation inhibits proliferation and enhances cisplatin sensitivity in cervical adenocarcinoma cells by regulating Bcl-2 and caspase-3, mediates apoptosis in bladder cancer cells and also reduces resistance of Fluorouracil (5-FU) in the treatment of cancers. Carbamoyl phosphate synthetase I (CPS1) is a ligase enzyme and a booster of energy production within the mitochondria metabolized urea cycle [40,41]. Overexpression of this protein in the present study indicates protein corona complication and results in poor metabolism of proteins and nitrogen, as well as high levels of ammonia production. Integrin beta-1 is also upregulated in our investigation, and its mode of action was triggered by the Fe_2_C NP stress, which controls the membrane receptors involved in cell adhesion [42] and recognition in a variety of processes, including hemostasis, tissue repair, immune response and metastatic diffusion of tumor cells, which is a therapeutic approach for reduction in cancer cells. However, the downregulation of CPS1 is considered the major player in triggering HCC in nearly 75% of cases. This involves DNA methylation, loss of cellular homeostasis and genetic mutations, along with genetic instability. Pyrroline-5-carboxylate reductase 1 (PYCR1) is an important protein which regulates the generation of NADP (+) in cells and involves oxidative stress in cell response [43,44]. The protein forms a homopolymer and localizes to the mitochondrion. In the current study, overexpression of this gene seems to be a property of rapidly dividing cells and enhanced their direct link to oncogenesis, as reported previously [45,46]. However, the downregulation of this protein causes it to act as a suppressor of cell proliferation and encourages cell apoptosis for potential therapeutic application over the tumor. Therefore, the particle treatment provides an indirect prognostic tool for the downregulation of oncogenic proteins for various and specific carcinomas. 

Upregulated CW protein ribosomal protein S25 plays a role in general protein synthesis; however, the RPS25 gene is non-essential for cellular viability in budding yeast and in select mammalian cell lines, implying that it is not essential for eukaryotic protein synthesis [47,48]. In this investigation, overexpression of this protein under NP stress might alter the expression level effects on protein synthesis. Heat shock 70 kDa protein 8, also known as heat shock cognate 71 kDa protein, is mainly involved in the regulation of the nuclear accumulation of cyclin D1, which is a key player in NPs the G1 to S phase cell cycle transition [49,50]. It plays a pivotal role in the protein quality control system, ensuring the correct folding of proteins, the re-folding of misfolded proteins and the control of the targeting of proteins for subsequent degradation [51,52]. In addition, RPS25 upregulation promotes c-Myc expression by both translational control and transcription mechanisms that ultimately promote renal cancer. Thioredoxin-dependent peroxide reductase, a mitochondrial enzyme in a family of antioxidant enzymes, is responsible for the regulation of cellular proliferation, differentiation and antioxidant functions. Differential expression of this protein due to NP stress is responsible for changes in the cell cycle signaling and other metabolic activities [53,54]. This enzyme is implicated in several cancers, including prostate, breast, colorectal and lung cancer. It can also be a valuable biomarker for the diagnosis and assessment of hepatocellular carcinoma. Serum peroxiredoxin3 is a useful biomarker for early diagnosis and assessment of the prognosis of hepatocellular carcinoma. As reported, the overexpressed thioredoxin-dependent peroxide reductase regulates the activity of DNA-binding proteins that include Jun/Fos and nuclear factor-κB, which promote oncogenic pathways. Further, the real-time quantitative RT-PCR analysis identified overexpressed thioredoxin-dependent peroxide reductase as a key player in oral cancer mortality. Ras-related protein Rab-7a is involved in endocytosis, which is a process that brings substances into a cell [55,56]. The process of endocytosis works by folding the cell membrane around a substance outside of the cell (for example a protein) and then forming a vesicle. The overexpressed Ras-related protein Rab-7a significantly triggers cell proliferation in the body and transforms prostate cancer. In addition, this acts as a biomarker for the early detection of cancer. Palmitoylome profiling indicates that androgens regulate the palmitoylation of α-tubulin in prostate-cancer-derived LNCaP cells and supernatants. Thereby, the overexpression of Rab-7a also contributes to the proliferation, invasion and xenograft tumor development of breast cancer cells (knockdown of Rab7a suppresses the proliferation, migration and xenograft tumor growth of breast cancer cells). Ribosomal protein L21 (RPL21 60S) is a protein which catalyzes the protein synthesis process [57]. Studies on the decreased expression of ribosomal proteins in human age-related cataracts suggest that modulation of protein synthesis and/or other functions mediated by ribosomal proteins are associated with age-related cataracts, whereas overexpressed RPL21 in pancreatic cancer results in discrete subsets of cellular proteins that are key promoters of PC cell proliferation. According to studies, RPL21 triggers colorectal cancer, liver cancer and prostate cancer when overexpressed. Therefore, the overexpressed protein significantly contributes to promoting cell proliferation and metastasis by initiating oncogenic inter-related cascades.

## 3. Materials and Methods

### 3.1. Green Synthesis of Iron Carbide NPs

Iron carbide NPs were synthesized by modifying those previously reported [27], where 1 g of iron nitrate was mixed with 1 g of glucose and 3 g of glycine into 200 mL of distilled water. The plant extract was made by mixing 10–20 g of finely refined powder of *Withania* with 300 mL of distilled water at 90 °C for 1 h and the filtrate was obtained by filtering the solution through filter paper. Then, 100 mL of iron nitrate solution was mixed with 100 mL of plant extract. The mixture was heated on a hot plate to 30 °C until the black gelatinous mass was obtained, confirming the successful synthesis of Fe_2_C NPs. The gelatinous product was mixed with 1 mL of ethanol and incubated at 100 °C for 24 h until the NPs were properly dried up and converted into powder for next use. Note: the information about the materials used here is provided in the Supporting Information.

### 3.2. Functionalization of Fe_2_C NPs

The Fe_2_C NPs were surface coated using our previously reported method with slight modification [16]. The iron carbide NPs were placed in 300 mL of ethanol and ammonia and mixed using a magnetic stirrer at a pH of 8.5–9.5. Later, N(trimethoxysilylpropyl) Ethylene Diamine-triaceticacid-trisodium salt was added slowly and a pH of ~3 was set using HCl; the mixture was then incubated for 15 h. The resulted Fe_2_C@COOH NPs were obtained for further characterization (provided in Supporting Information) and application. 

### 3.3. Cell Culture and NP Incubation

Initially, HeLa cells were grown in Petri plates by adding Dulbecco’s Modified Eagle Medium (DMEM) supplemented with 15% fetal bovine serum (FBS). The cells were then kept in a 5% CO_2_ incubator for 48 h. The samples include untreated HeLa cells, and HeLa cells treated with Fe_2_C NPs and Fe_2_C@COOH NPs. 

### 3.4. Sample Preparation

Fe_2_C NPs with and without modification were added with agar media in the respective HeLa cell group for 48 h. Trypsin was used to eradicate cells from the surface of Petri plates with NPs. Tris, 3 M urea (5 mL), 1 M thiourea (5 mL), 2% 3-[(3-cholamidopropyl) dimethylammonio]-1-propanesulfonate (CHAPS) (15 mL), 5 mM 1–4 dithioerythritol and 0.5 mM Ethylenediaminetetraacetic acid protein inhibitor were added to avoid any alteration to the system. The samples were centrifuged slightly to separate NPs from the cell. Separated NPs were centrifuged at high speed to separate protein from the surface of NPs. The separated protein content was finally measured using the Bradford reagent.

### 3.5. Protein Digestion and Peptide Formation

The proteins from the surface of NPs were denatured by 50 μL of 6 M urea and then with 6 μL of 100 mM 2.2-Dithiodipyridine (DTP) which lyophilized with 6 h incubation at 37 °C. The samples were then kept in an alkaline environment by adding 30 μL of 100 mM iodocamide for 24 h in dark. Finally adding 600 μL of 50 mM NH_4_CO_3_ (pH 8.2) and 40 μL of trypsin (0.5 mg/mL) resulted in the formation of peptides, which was continued for 20 h at room temperature.

### 3.6. Cation Exchange Fractionation

The proteins isolated from the surface of NPs were added at a flow rate of 0.5 mL/min to a 250 mm column at set conditions according to Agilent 300-A SCX 4.6 mm [29]. The mobile phase of solvent A consisted of 15 mM ammonia format, 25% acetonitrile at pH of 3, while solvent B was composed of 500 mM ammonium format, 25% acetonitrile at a pH of 6.8. A 3 to 4% linear gradient of B separated peptides over 50 min at 280 nm. The fractions of protein peptides were collected after every 2 min and stored at 80 °C.

### 3.7. Liquid Chromatography–Mass Spectrometry/Mass Spectrometry Analysis

The column C18 (Column Technology Inc., Fremont, CA, USA) was used to elute the samples with a linear acetonitrile gradient elution from 100% solvent for liquid chromatography–mass spectrometry/mass spectrometry (LC-MS/MS) analysis or Agilent electrospray–ion trap (ESI-Trap) mass spectrometry analysis. Both solvent A consisting of 70% water-based mixture with 0.1% formic acid, and solvent B consisting of 30% acetonitrile-based mixture with 0.1% formic acid, were kept in the column for 2 h with a flow rate of 2.01 mL/min [30]. When operating ESI-TOF and ESI-TROP analyzers, the nebulizer pressure was kept 25 psi. The drying gas (nitrogen) flow was 12 mL/min at 310 °C.

### 3.8. Data Analysis

The data analysis was carried out using Swiss-Prot protein database, from the Mascot (Matrix Science, Marylebone, UK) server (http://www.expasy.ch/sprot accessed on 27 June 2022). The protein identical probabilities were determined using Mascot protein scores, with identification confidence, using all related information, i.e., number of matches and coverage of protein sequence by matching peptides, as conducted in the previous method [30]. Only statistically significant Mascot score results were included in the data analysis or the quantification of HPLC/ESI-TOF data. 

## 4. Conclusions

In summary, we identified 142 differentially expressed proteins in response to Fe_3_C NPs in HeLa cells. The carboxyl functionalized COOH tailoring enhanced their biocompatibility and stability, which helps to identify potential biomarkers at early stages of diseases. The differentially expressed proteins were further sub-classified into 88 common and 54 unique proteins and arranged in the proteomics table. These proteins were further categorized on behalf of their regularity, locality, molecular functionality, molecular masses and STRING analysis, which leads to targeting the specific range of proteins involved in metabolic pathways and molecular processing. This will help gain insight into novel possibilities in the future regarding protein fingerprinting by surface modifications, which provides useful identification of potential biomarkers for early detection of diseases such as carcinomas, as well as will help advance nanomedicine and biomedical applications.

## Data Availability

The data presented in this study are available in the Supplementary Material.

## Supplementary Materials

The following supporting information can be downloaded at: https://www.mdpi.com/article/10.3390/molecules27185754/s1, Figure S1: Ultraviolet-visible (UV-vis) and Fourier transform infrared (FTIR) spectra of coated and uncoated NPs; Table S1: Total differential proteins of HeLa cells around Fe2C NPs; Table S2: Tame-type proteins (CW); Table S3: Mild-type proteins (CY); Table S4: Wild-type proteins (WY); Table S5: Common proteins in CW-CY; Table S6: Common proteins in CW-WY; Table S7: Common proteins in CY-WY; Table S8: Common proteins in CWY; Table S9: Unique proteins in CW; Table S10: Unique proteins in CY; Table S11: Unique proteins in WY.

## Sample Availability

Samples of the compounds are not available from the authors.

## References

[1] Serban K.A., Pratte K.A., Bowler R.P. (2021). Protein Biomarkers for COPD Outcomes. Chest.

[2] Hasan M., Yang W., Ju Y., Chu X., Wang Y., Deng Y., Mahmood N., Hou Y. (2017). Biocompatibility of iron carbide and detection of metals ions signaling proteomic analysis via HPLC/ESI-Orbitrap. Nano Res..

[3] Kaur M., Tiwari S., Jain R. (2020). Protein based biomarkers for non-invasive COVID-19 detection. Sens. Bio-Sensing Res..

[4] Ueda K. (2018). A proteome-wide catalog of extracellular vesicles, toward development of cancer liquid biopsy diagnostics. Cancer Sci..

[5] Hasan M., Mustafa G., Iqbal J., Ashfaq M., Mahmood N. (2018). Quantitative proteomic analysis of HeLa cells in response to biocompatible Fe2C@C nanoparticles: 16O/18O-labelling & HPLC-ESI-orbit-trap profiling approach. Toxicol Res..

[6] Zafar A., Jabbar M., Manzoor Y., Gulzar H., Hassan S.G., Nazir M.A., Ain-ul-Haq, Mustafa G., Sahar R., Masood A. (2020). Quantifying Serum Derived Differential Expressed and Low Molecular Weight Protein in Breast Cancer patients. Protein Pept. Lett..

[7] Iqbal J., Li W., Hasan M., Liu K., Awan U., Saeed Y., Zhang Y., Khan A.M., Shah A., Qing H. (2014). Differential expression of specific cellular defense proteins in rat hypothalamus under simulated microgravity induced conditions: Comparative proteomics. Proteomics.

[8] Lu C., Urban M.W. (2018). Stimuli-responsive polymer nano-science: Shape anisotropy, responsiveness, applications. Prog. Polym. Sci..

[9] García-álvarez R., Vallet-Regí M. (2021). Hard and soft protein corona of nanomaterials: Analysis and relevance. Nanomaterials.

[10] Kopac T. (2021). Protein corona, understanding the nanoparticle–protein interactions and future perspectives: A critical review. Int. J. Biol. Macromol..

[11] Saleh D.A., Shimoni O., Sosnik A. (2017). Novel core-corona hybrid nanomaterials based on the conjugation of amphiphilic polymeric diblocks to the surface of multifunctional nanodiamond anchors. Mater. Today Chem..

[12] Visalakshan R.M., García L.E.G., Benzigar M.R., Ghazaryan A., Simon J., Mierczynska-Vasilev A., Michl T.D., Vinu A., Mailänder V., Morsbach S. (2020). The Influence of Nanoparticle Shape on Protein Corona Formation. Small..

[13] Blume J.E., Manning W.C., Troiano G., Hornburg D., Figa M., Hesterberg L., Platt T.L., Zhao X., Cuaresma R.A., Everley P.A. (2020). Rapid, deep and precise profiling of the plasma proteome with multi-nanoparticle protein corona. Nat. Commun..

[14] Xu L., Xu M., Wang R., Yin Y., Lynch I., Liu S. (2020). The Crucial Role of Environmental Coronas in Determining the Biological Effects of Engineered Nanomaterials. Small.

[15] Sebak A.A., Gomaa I.E.O., Elmeshad A.N., Farag M.H., Breitinger U., Breitinger H.G., Abdelkader M.H. (2020). Distinct proteins in protein corona of nanoparticles represent a promising venue for endogenous targeting – part ii: In vitro and in vivo kinetics study. Int. J. Nanomed..

[16] Hasan M., Gulzar H., Zafar A., Haq A., Mustafa G., Tariq T., ul Khalid A., Mahmmod A., Shu X., Mahmood N. (2021). Multiplexing surface anchored functionalized iron carbide nanoparticle: A low molecular weight proteome responsive nano-tracer. Colloids Surf. B Biointerfaces.

[17] Albakova Z., Siam M.K.S., Sacitharan P.K., Ziganshin R.H., Ryazantsev D.Y., Sapozhnikov A.M. (2021). Extracellular heat shock proteins and cancer: New perspectives. Transl. Oncol..

[18] Ignjatovic V., Geyer P.E., Palaniappan K.K., Chaaban J.E., Omenn G.S., Baker M.S., Deutsch E.W., Schwenk J.M. (2019). Mass spectrometry-based plasma proteomics: Considerations from sample collection to achieving translational data. bioRxiv.

[19] Strojan K., Leonardi A., Bregar V.B., Križaj I., Svete J., Pavlin M. (2017). Dispersion of nanoparticles in different media importantly determines the composition of their protein corona. PLoS ONE.

[20] Breznica P., Koliqi R., Daka A. (2020). A review of the current understanding of nanoparticles protein corona composition. Med. Pharm. Rep..

[21] Otun K.O., Yao Y., Liu X., Hildebrandt D. (2021). Synthesis, structure, and performance of carbide phases in Fischer-Tropsch synthesis: A critical review. Fuel.

[22] Zhang Z., Yin H., Yu G., He S., Kang J., Liu Z., Cheng K., Zhang Q., Wang Y. (2021). Selective hydrogenation of CO_2_ and CO into olefins over Sodium- and Zinc-Promoted iron carbide catalysts. J. Catal..

[23] Zafar A., Tariq T., Hasan M., Nazar M., Rasheed M.N., Mahmood N., Shu X. (2021). Green-maturation of Cobalt-Oxide nano-sponges for reinforced bacterial apoptosis. Colloids Interface Sci. Commun..

[24] Hasan M., Ullah I., Zulfiqar H., Naeem K., Iqbal A., Gul H., Ashfaq M., Mahmood N. (2018). Biological entities as chemical reactors for synthesis of nanomaterials: Progress, challenges and future perspective. Mater. Today Chem..

[25] Hasan M., Zafar A., Shahzadi I., Luo F., Hassan S.G., Tariq T., Zehra S., Munawar T., Iqbal F., Shu X. (2020). Fractionation of biomolecules in Withania coagulans extract for bioreductive nanoparticle synthesis, antifungal and biofilm activity. Molecules.

[26] Saif M.S., Zafar A., Waqas M., Hassan S.G., Haq A., Tariq T., Batool S., Dilshad M., Hasan M., Shu X. (2021). Phyto-reflexive Zinc Oxide Nano-Flowers synthesis: An advanced photocatalytic degradation and infectious therapy. J. Mater. Res. Technol..

[27] Qasim S., Zafar A., Saif M.S., Ali Z., Nazar M., Waqas M., Haq A.U., Tariq T., Hassan S.G., Iqbal F. (2020). Green synthesis of iron oxide nanorods using Withania coagulans extract improved photocatalytic degradation and antimicrobial activity. J. Photochem. Photobiol. B Biol..

[28] Zarei M., Aalaie J. (2018). Profiling of nanoparticle–protein interactions by electrophoresis techniques. Anal. Bioanal. Chem..

[29] Iqbal J., Li W., Hasan M., Li Y.J., Ullah K., Yun W., Awan U., Qing H., Deng Y. (2014). Distortion of homeostatic signaling proteins by simulated microgravity in rat hypothalamus: A^16^O/^18^O-labeled comparative integrated proteomic approach. Proteomics.

[30] Iqbal J., Li W., Ullah K., Hasan M., Linna G., Awan U., Zhang Y., Batool S., Qing H., Deng Y. (2013). Study of rat hypothalamic proteome by HPLC/ESI ion trap and HPLC/ESI-Q-TOF MS. Proteomics.

[31] Tenzer S., Docter D., Kuharev J., Musyanovych A., Fetz V., Hecht R., Schlenk F., Fischer D., Kiouptsi K., Reinhardt C. (2013). Rapid formation of plasma protein corona critically affects nanoparticle pathophysiology. Nat. Nanotechnol..

[32] Corchero J.L., Villaverde A. (2009). Biomedical applications of distally controlled magnetic nanoparticles. Trends Biotechnol..

[33] Lu C., Zhao H., Luo C., Lei T., Zhang M. (2020). Knockdown of ferritin heavy chain (FTH) inhibits the migration of prostate cancer through reducing S100A4, S100A2, and S100P expression. Transl. Cancer Res..

[34] Cutolo M., Gotelli E., Montagna P., Tardito S., Paolino S., Pizzorni C., Sulli A., Smith V., Soldano S. (2021). Nintedanib downregulates the transition of cultured systemic sclerosis fibrocytes into myofibroblasts and their pro-fibrotic activity. Arthritis Res. Ther..

[35] Zhou Y., Wang Y., Wu S., Yan Y., Hu Y., Zheng Z., Li J., Wu W. (2020). Sulforaphane-cysteine inhibited migration and invasion via enhancing mitophagosome fusion to lysosome in human glioblastoma cells. Cell Death Dis..

[36] Elsaadi S., Steiro I., Abdollahi P., Vandsemb E.N., Yang R., Slørdahl T.S., Rø T.B., Menu E., Sponaas A.M., Børset M. (2021). Targeting phosphoglycerate dehydrogenase in multiple myeloma. Exp. Hematol. Oncol..

[37] Li M., Wu C., Yang Y., Zheng M., Yu S., Wang J., Chen L., Li H. (2021). 3-Phosphoglycerate dehydrogenase: A potential target for cancer treatment. Cell. Oncol..

[38] Ali A., al Dhahouri N., Almesmari F.S.A., Fathalla W.M., al Jasmi F. (2021). Characterization of etfdh and phgdh mutations in a patient with mild glutaric aciduria type ii and serine deficiency. Genes.

[39] Zhang Y., Yu H., Zhang J., Gao H., Wang S., Li S., Wei P., Liang J., Yu G., Wang X. (2021). Cul4A-DDB1–mediated monoubiquitination of phosphoglycerate dehydrogenase promotes colorectal cancer metastasis via increased S-adenosylmethionine. J. Clin. Investig..

[40] Singh C., Benos A., Grenell A., Tran V., Hanna D., Anand-Apte B., Brunengraber H., Sears J.E. (2021). The urea cycle is transcriptionally controlled by hypoxia-inducible factors. bioRxiv.

[41] Zhang M., Wang S., Sun L., Gan L., Lin Y., Shao J., Jiang H., Li M. (2021). Ammonia induces changes in carbamoyl phosphate synthetase I and its regulation of glutamine synthesis and urea cycle in yellow catfish Pelteobagrus fulvidraco. Fish Shellfish Immunol..

[42] Sun Q., Kanehira K., Taniguchi A. (2018). PEGylated TiO2 nanoparticles mediated inhibition of cell migration via integrin beta 1. Sci. Technol. Adv. Mater..

[43] Sang S., Zhang C., Shan J. (2019). Pyrroline-5-Carboxylate Reductase 1 Accelerates the Migration and Invasion of Nonsmall Cell Lung Cancer In Vitro. Cancer Biotherapy Radiopharm..

[44] Cai F., Miao Y., Liu C., Wu T., Shen S., Su X., Shi Y. (2017). Pyrroline-5-carboxylate reductase 1 promotes proliferation and inhibits apoptosis in non-small cell lung cancer. Oncol. Lett..

[45] Xiao S., Li S., Yuan Z., Zhou L. (2020). Pyrroline-5-carboxylate reductase 1 (PYCR1) upregulation contributes to gastric cancer progression and indicates poor survival outcome. Ann. Transl. Med..

[46] Ding J., Kuo M.L., Su L., Xue L., Luh F., Zhang H., Wang J., Lin T.G., Zhang K., Chu P. (2017). Human mitochondrial pyrroline-5-carboxylate reductase 1 promotes invasiveness and impacts survival in breast cancers. Carcinogenesis.

[47] Takeuchi I., Nasukawa T., Sugimoto R., Takemura-Uchiyama I., Murakami H., Uchiyama J. (2021). Analyses of propagation processes of Staphylococcus aureus bacteriophages S13′ and S25-3 in two different taxonomies by definitive screening design. Virus Res..

[48] Mohapatra B.R. (2020). Characterization of β-mannanase extracted from a novel Streptomyces species Alg-S25 immobilized on chitosan nanoparticles. Biotechnol. Biotechnol. Equip..

[49] Solarz A., Majcher-Maślanka I., Kryst J., Chocyk A. (2021). A Search for Biomarkers of Early-life Stress-related Psychopathology: Focus on 70-kDa Heat Shock Proteins. Neuroscience.

[50] Orfanelli T., Giannopoulos S., Zografos E., Athanasiou A., Bongiovanni A.M., Doulaveris G., Moo T.A., LaPolla D., Bakoyiannis C.N., Theodoropoulos G.E. (2021). Alterations of the 70 kDa heat shock protein (HSP70) and sequestosome-1 (p62) in women with breast cancer. Sci. Rep..

[51] González-Ruiz L., González-Moles M.Á., González-Ruiz I., Ruiz-Ávila I., Ayén Á., Ramos-García P. (2020). An update on the implications of cyclin D1 in melanomas. Pigment Cell Melanoma Res..

[52] Montalto F.I., de Amicis F. (2020). Cyclin D1 in Cancer: A Molecular Connection for Cell Cycle Control, Adhesion and Invasion in Tumor and Stroma. Cells.

[53] Maqbool I., Ponniresan V.k., Govindasamy K., Prasad N.R. (2020). Understanding the survival mechanisms of Deinococcus radiodurans against oxidative stress by targeting thioredoxin reductase redox system. Arch Microbiol..

[54] Pourmohammadi K., Abedi E. (2021). Enzymatic modifications of gluten protein: Oxidative enzymes. Food Chem..

[55] Yang Y., Zhu Y., Li X., Zhang X., Yu B. (2021). Identification of potential biomarkers and metabolic pathways based on integration of metabolomic and transcriptomic data in the development of breast cancer. Arch. Gynecol. Obstet..

[56] Li M., Yu W., Ke X., Ye P., Peng J., Li H. (2021). Downregulation of rab7 and caveolin-1 increases mmp-2 activity in renal tubular epithelial cells under hypoxic conditions. Open Med..

[57] Xie M., Kobayashi I., Kiyoshima T., Nagata K., Ookuma Y., Fujiwara H., Sakai H. (2009). In situ expression of ribosomal protein L21 in developing tooth germ of the mouse lower first molar. J. Mol. Histol..

