# Supplementary material for: Trident Nano-Indexing the Proteomics Table: Next-Version Clustering of Iron Carbide NPs and Protein Corona
Source: Molecules. 2022 Sep 6;27(18):5754. doi: 10.3390/molecules27185754 (PMC9500999; doi:10.3390/molecules27185754)
Supplement: Supplementary file 1 [file molecules-27-05754-s001.zip › molecules-1860827-supplementary.pdf]

Supporting information

Trident Nano-Indexing the Proteomics Table: Next-Version Clustering of Iron Carbide NPs and Protein Corona

Murtaza Hasan [1,2,*], Ayesha Zafar [2,3], Maryum Jabbar [2], Tuba Tariq [2], Yasmeen Manzoor [2], Muhammad Mahmood Ahmed [2], Shahbaz Gul Hassan [4], Xugang Shu [1,*] and Nasir Mahmood [5,*]

[1] School of Chemistry and Chemical Engineering, Zhongkai University of Agriculture and Engineering, Guangzhou 510225, China

[2] Department of Biotechnology, The Islamia University of Bahawalpur, Bahawalpur 63100, Pakistan;

[3] Department of Biomedical Engineering, College of Future Technology, Peking University, Beijing 100871, China

[4] College of Information Science and Engineering, Zhongkai University of Agriculture and Engineering, Guangzhou 510225, China

[5] School of Science, RMIT University, Victoria 3000, Australia

Corresponding Author

Dr Murtaza Hasan (murtaza@zhku.edu.cn)

Professor Xugang Shu (xgshu@21cn.com)

Dr Nasir Mahmood (nasir.mahmood@rmit.edu.au)

Materials and Methods

Materials

Iron nitrate ($Fe(NO_3)_3.9H_2O$), glycine ($C_2H_5NO_2$), and glucose($C_6H_{12}O_6.H_2O$) were purchased from Sigma-Aldrich. *Withania* plant extract as Bioreducing agent and Distilled water and 99% ethanol were used. HeLa cell line was provided by Bahawal Victoria Hospital Bahawalpur.

Physical Characterization

The Ultraviolet-visible (UV-vis) absorption spectra of green synthesized iron carbide NPs were recorded using a S22PC spectrophotometer (China) with wavelength range of 200-800 nm. For investigating about the presence of reducing biomolecules groups and interaction of green synthesized iron carbide with plant extract, the Fourier transform infrared (FTIR) spectra was performed using the instrument Spectrum100 model (Perkin Elmer, USA). To observe and detect the microscopic morphology, particle size distribution for iron carbide NPs and microscopic analysis was performed on a transmission electron microscope (TEM, Talos 200s, FEI, USA) equipped.

Results

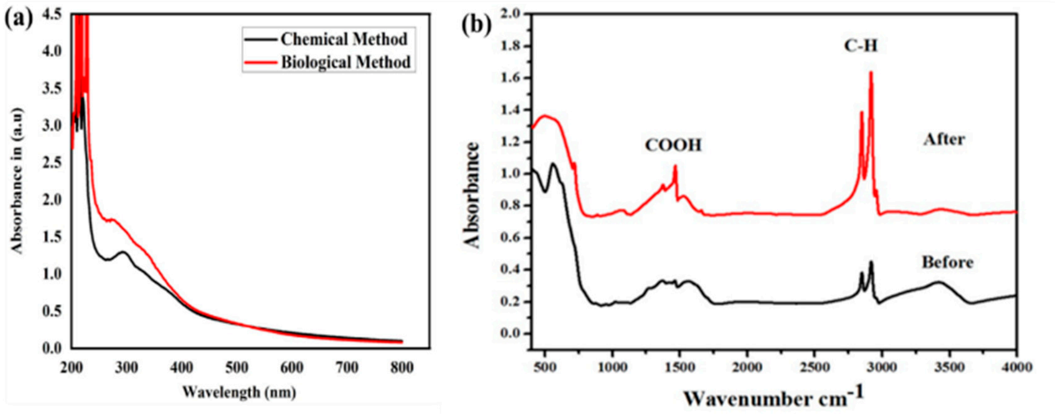

Figure S1. Ultraviolet-visible (UV-vis) and Fourier transform infrared (FTIR) spectra of coated and uncoated NPs.

Table S1. Total differential proteins of HeLa cells around Fe_2C NPs.

	**Code**	**Gene name**	**Protein name**	**Ratio**	**Mol. masses kDa**	**Location**	**Molecular Function**	**Biological function**
1.	P26447	S100A4	S100-A4	1.71	11.729	Extracellular region or secreted<br>Nucleus	RNA binding	positive regulation of I-kappaB kinase/NF-kappaB signaling
2.	P01891	HLA-A	1A68	1.80	40.909	ER<br>PM	RNA binding	regulation of immune response
3.	P18669	PGAM1	Phosphoglycerate mutase 1	2.00	28.804	Cytosol<br>ER	phosphoglycerate mutase activity	glycolytic process
4.	P32119	PRDX2	Peroxiredoxin-2	2.00	21.892	Cytoplasm<br>cytosol	antioxidant activity	cell redox homeostasis
5.	O43175	PHGDH	D-3-phosphoglycerate dehydrogenase	2.00	56.651	Cytosol<br>ER	phosphoglycerate dehydrogenase activity	Brain development
6.	P69905	HBA1	Hemoglobin subunit alpha	2.11	15.258	Cytosol<br>ER	Heme binding	Oxygen transport

7.	P60842	EIF4A1	Eukaryotic initiation factor 4A-I	2.50	46.154	Cytosol<br><br>ER	RNA binding	cytoplasmic translational initiation
8.	Q07955	SRSF1	Serine/arginine-rich splicing factor 1	3.50	27.745	Nucleus<br><br>Cytoplasm	RNA binding	alternative mRNA splicing, via spliceosome
9.	Q13162	PRDX4	Peroxiredoxin-4	0.29	30.540	ER<br><br>cytoplam	thioredoxin peroxidase activity	cell redox homeostasis
10.	P62424	RPL7A	60S ribosomal protein L7a	0.29	29.996	Cytosol<br><br>Nucleus	RNA binding	Translation
11.	P23284	PPIB	Peptidyl-prolyl cis-trans isomerase B	0.29	23.743	ER<br><br>Extracellular region	RNA binding	Bone development
12.	Q07065	CKAP4	Cytoskeleton-associated protein 4	0.30	66.022	Cytoskeleton<br><br>PM	RNA binding	Post translation protein modification
13.	Q96AG4	LRRC59	Leucine-rich repeat-containin g protein 59	0.33	34.930	Nucleus<br><br>ER	RNA binding	-
14.	P16401	HIST1H1B	Histone H1.5	0.36	22.580	Nucleus<br><br>Chromosome	RNA binding	Chromatin organization
15.	Q8NBS9	TXNDC5	Thioredoxin domain-containing protein 5	0.38	47.629	ER<br><br>Extracellular region	isomerase activity	apoptotic cell clearance
16.	P04792	HSPB1	Heat shock protein beta-1	0.39	22.783	Cytoskeleton<br><br>Nucleus	RNA binding	anterograde axonal protein transport
17.	Q14697	GANAB	Neutral alpha-glucosidase AB	0.40	106.874	ER<br><br>Golgi apparatus	RNA binding	N-glycan processing
18.	P30101	PDIA3	Protein disulfide-isomerase A3	0.40	56.782	ER<br><br>Endosome	RNA binding	Protein folding
19.	P36578	RPL4	60S ribosomal protein L4	0.40	47.697	Cytosol<br><br>ER	RNA binding	Translation
20.	P35579	MYH9	Myosin-9	0.43	226.532	Cytoskeleton	RNA binding	actin cytoskeleton reorganization

						cytosol		
21.	Q15084	PDIA6	Protein disulfide-isomerase A6	0.45	48.121	ER<br><br>PM	peptide disulfide oxidoreductase activity	Protein folding
22.	P00966	ASS1	Argininosuccinate synthase	0.46	46.530	Cytosol<br><br>ER	RNA binding	arginine biosynthetic process
23.	P31327	CPS1	Carbamoyl-phosphate synthase [ammonia], mitochondrial	0.49	164.939	Mitochondria<br><br>Nucleus	ATP binding	carbamoyl phosphate biosynthetic process
24.	O43852	CALU	Calumenin	0.50	37.107	ER<br><br>Golgi apparatus	calcium ion binding	cellular protein metabolic process
25.	P10606	COX5B	Cytochrome c oxidase subunit 5B, mitochondrial	0.50	13.696	Mitochondria	cytochrome-c oxidase activity	mitochondrial ATP synthesis coupled proton transport
26.	P05556	ITGB1	Integrin beta-1	0.50	88.415	Endosome<br><br>PM	Actin binding	B cell differentiation
27.	P20700	LMNB1	Lamin-B1	0.50	66.408	Nucleus<br><br>Cytoskeleton	structural molecule activity	interleukin-12-mediated signaling pathway
28.	P32322	PYCR1	Pyrroline-5-carboxylate reductase 1, mitochondrial	0.50	33.361	Mitochondria	identical protein binding	L-proline biosynthetic process
29.	O75340	PDCD6	Programmed cell death protein 6	0.50	21.868	Nucleus<br><br>endosome	Calcium ion binding	COPII vesicle coating
30.	Q15149	PLEC	Plectin	0.50	531.791	Cytoskeleton<br><br>Cytosol	RNA binding	hemidesmosome assembly
31.	P39023	RPL3	60S ribosomal protein L3	0.50	46.109	Nucleus<br><br>Cytosol	RNA binding	Translation
32.	P62851	RPS25	40S ribosomal protein S25	0.50	13.742	Cytosol<br><br>Extracellular region	RNA binding	Translation

33.	P61247	RPS3A	40S ribosomal protein S3a	0.50	29.945	Nucleus<br><br>ER	RNA binding	Translation
34.	P08670	VIM	Vimentin	0.53	53.652	Cytoskeleto n<br><br>Nucleus	double-stranded RNA binding	positive regulation of translation
35.	P61006	RAB8A	Ras-related protein Rab-8A	0.56	23.668	Cytoskeleto n<br><br>Endosome	GTP binding	Rab protein signal transduction
36.	P18124	RPL7	60S ribosomal protein L7	0.56	29.226	Cytosol<br><br>nucleus	DNA binding RNA binding	Translation
37.	P62280	RPS11	40S ribosomal protein S11	0.57	18.431	Cytosol<br><br>Extracellula r region	RNA binding	Translation
38.	P11142	HSPA8	Heat shock cognate 71 kDa protein	0.58	70.898	Plasma membrane<br><br>Nucleus	ATP binding	ATP metabolic process
39.	P15311	EZR	Ezrin	0.60	69.413	Cytoskeleto n<br><br>PM	ATPase binding	actin cytoskeleton reorganization
40.	Q99714	HSD17B10	3-hydroxyacyl-CoA dehydrogena se type-2	0.60	26.923	Mitochondr ia<br><br>PM	3-hydroxyacyl-CoA dehydrogenase activity	lipid metabolic process
41.	P55209	NAP1L1	Nucleosome assembly protein 1-like 1	0.60	45.374	Nucleus<br><br>Cytoplasm	RNA binding	DNA replication
42.	P08238	HSP90AB1	Heat shock protein HSP 90-beta	0.62	83.264	PM<br><br>Extracellula r region	ATP binding	Protein folding
43.	P80723	BASP1	Brain acid soluble protein 1	0.63	22.693	PM<br><br>Extracellula r region	protein domain specific binding	diaphragm development
44.	P02545	LMNA	Prelamin-A/C	0.63	74.139	Nucleus<br><br>cytoskeleto n	identical protein binding	cellular protein localization
45.	P07237	P4HB	Protein disulfide-isomerase	0.63	57.116	PM<br><br>ER	RNA binding	Protein folding

46.	P09923	ALPI	Intestinal-type alkaline phosphatase	0.63	56.812	PM<br><br>Extracellular region	alkaline phosphatase activity	Dephosphorylation
47.	P05186	ALPL	Alkaline phosphatase, tissue-nonspecific isozyme	0.63	57.305	PM<br><br>Extracellular region	alkaline phosphatase activity	skeletal system development
48.	P38646	HSPA9	Stress-70 protein, mitochondrial	0.65	73.680	Mitochondria<br><br>Nucleus	ATP binding	cellular response to heat
49.	P14618	PKM	Pyruvate kinase PKM	0.65	57.937	Mitochondrio<br><br>nucleus	ATP binding	ATP biosynthetic process
50.	P0DMV8	HSPA1A	Heat shock 70 kDa protein 1A	0.65	70.052	Cytoskeleton<br><br>Nucleus	ATP binding	ATP metabolic process
51.	P40926	MDH2	Malate dehydrogenase, mitochondrial	0.65	35.503	Mitochondria<br><br>Extracellular region	L-malate dehydrogenase activity	aerobic respiration
52.	P07355	ANXA2	Annexin A2	0.65	38.604	Extracellular region or secreted<br><br>Cytosol	RNA binding	Angiogenesis
53.	P49411	TUFM	Elongation factor Tu, mitochondrial	0.67	49.542	Mitochondria<br><br>Extracellular region	GTP binding	mitochondrial translational elongation
54.	P35268	RPL22	60S ribosomal protein L22	0.67	14.787	Cytosol<br><br>Extracellular region	RNA binding	Translation
55.	P04843	RPN1	Dolichyl-diphosphooligosaccharide--protein glycosyltransferase subunit 1	0.67	68.569	ER<br><br>Cytosol	RNA binding	cellular protein modification process
56.	P45880	VDAC2	Voltage-dependent anion-selective	0.67	31.567	Mitrochondria	voltage-gated anion channel activity	anion transport

			channel protein 2					
57.	P08133	ANAX6	Annexin A6	1.75	75.873	Cytoplasm<br><br>Endosome	GTP binding	apoptotic signaling pathway
58.	P00533	EGFR	Epidermal growth factor receptor	2.33	134.277	E.R<br><br>PM	ATP binding	activation of phospholipase A2 activity by calcium-mediated signaling
59.	P16403	HIST1H1C	Histone H1.2	1.71	21.365	Nucleus<br><br>Chromosome	RNA binding	chromosome condensation
60.	P60842	EIF4A1	Eukaryotic initiation factor 4A-I	2.50	46.154	Cytosol<br><br>Extracellular region	RNA binding	cytoplasmic translational initiation
61.	P13667	PDIA4	Protein disulfide-isomerase A4	2.00	72.932	ER<br><br>Extracellular region	RNA binding	Protein folding
62.	P00558	PGK1	Phosphoglycerate kinase 1	3.00	44.615	Cytoplasm<br><br>Cytosol	ADP binding	cellular response to hypoxia
63.	P62249	RPS16	40S ribosomal protein S16	1.67	16.445	Cytosol<br><br>Extracellular region	RNA binding	Translation
64.	Q07955	SRSF1	Serine/arginine-rich splicing factor 1	2.50	27.745	Nucleus<br><br>Cytoplasm	DNA topoisomerase binding	alternative mRNA splicing, via spliceosome
65.	P27105	STOM	Erythrocyte band 7 integral membrane protein	2.00	31.731	Cytoskeleton<br><br>PM	RNA polymerase binding	protein homooligomerization
66.	Q09666	AHNAK	Neuroblast differentiation-associated protein AHNAK	0.37	629.101	Nucleus<br><br>Cytoskeleton	RNA binding	protein complex oligomerization
67.	P80723	BASP1	Brain acid soluble protein 1	0.63	22.693	PM<br><br>Cytoskeleton	protein domain specific binding	negative regulation of transcription, DNA-templated
68.	O43852	CALU	Calumenin	0.50	37.107	ER<br><br>Golgi apparatus	calcium ion binding	cellular protein metabolic process

69.	P61604	HSPE1	10 kDa heat shock protein, mitochondria	0.64	10.932	Mitochondria<br><br>Extracellular region	ATP binding	Protein folding
70.	P58107	EPPK1	Epiplakin	0.50	555.658	Cytoskeleton<br><br>PM	RNA binding	intermediate filament bundle assembly
71.	P38646	HSPA9	Stress-70 protein, mitochondrial	0.65	73.680	Nucleus<br><br>Mitochondria	ATP binding	Protein folding
72.	P16401	HIST1H1B	Histone H1.5	0.64	22.580	Nucleus	RNA binding	chromatin organization
73.	P33778	HIST1H2BB	Histone H2B type 1-B	0.51	13.950	Nucleus<br><br>Cytosol	DNA binding	nucleosome assembly
74.	P62807	HIST1H2BC	Histone H2B type 1-C/E/F/G/I	0.53	13.906	Nucleus<br><br>Cytosol	DNA binding	antibacterial humoral response
75.	Q99714	HSD17B10	3-hydroxyacyl-CoA dehydrogenase type-2	0.60	26.923	Mitochondria<br><br>PM	RNA binding	mitochondrial tRNA 3'-end processing
76.	P11279	LAMP1	Lysosome-associated membrane glycoprotein 1	0.40	44.882	Cytosol<br><br>Endosome	enzyme binding	Golgi to lysosome transport
77.	P55209	NAP1L1	Nucleosome assembly protein 1-like 1	0.40	45.374	Nucleus<br><br>Cytoplasm	RNA binding	DNA binding
78.	O75340	PDCD6	Programmed cell death protein 6	0.38	21.868	ER<br><br>Nucleus	calcium ion binding	COPII vesicle coating
79.	Q15084	PDIA6	Protein disulfide-isomerase A6	0.55	48.121	ER<br><br>PM	peptide disulfide oxidoreductase activity	Protein folding
80.	Q99623	PHB2	Prohibitin-2	0.62	33.296	Nucleus	amide binding	mitochondrion organization

						Mitochondria		
81.	P09923	ALP1	Intestinal-type alkaline phosphatase	0.63	56.812	PM<br><br>Extracellular region	alkaline phosphatase activity	Dephosphorylation
82.	P30048	PRDX3	Thioredoxin-dependent peroxide reductase, mitochondrial	0.50	27.693	Endosome<br><br>Mitochondria	alkyl hydroperoxide reductase activity	cell redox homeostasis
83.	P07737	PFN1	Profilin-1	0.56	15.054	Cytoskeleton<br><br>cytosol	RNA binding	protein stabilization
84.	P61026	RAB10	Ras-related protein Rab-10	0.58	22.541	Cytoskeleton<br><br>ER	GDP binding	Rab protein signal transduction
85.	P51149	RAB7A	Ras-related protein Rab-7a	0.60	23.490	Endosome<br><br>Cytosol	GDP binding	Rab protein signal transduction
86.	P26373	RPL13	60S ribosomal protein L13	0.63	24.261	Cytosol<br><br>ER	RNA binding	Translation
87.	P46778	RPL21	60S ribosomal protein L21	0.60	18.565	ER<br><br>Cytosol	RNA binding	Translation
88.	P83731	RPL24	60S ribosomal protein L24	0.67	17.779	Cytosol<br><br>ER	RNA binding	Translation
89.	P36578	RPL4	60S ribosomal protein L4	0.40	47.697	Cytosol<br><br>ER	RNA binding	Translation
90.	P18124	RPL7	60S ribosomal protein L7	0.44	29.226	Cytosol<br><br>nucleus	DNA binding	Translation
91.	P62424	RPL7A	60S ribosomal protein L7a	0.50	29.996	Cytosol<br><br>nucleus	RNA binding	Translation
92.	P52815	MRPL12	39S ribosomal protein L12, mitochondrial	0.67	21.348	Mitochondria	RNA binding	mitochondrial transcription
93.	P62280	RPS11	40S ribosomal protein S11	0.57	18.431	Cytosol<br><br>Extracellular region	RNA binding	Translation

94.	P62244	RPS15A	40S ribosomal protein S15a	0.67	14.840	Cytosol<br>Extracellular region	RNA binding	Translation
95.	P51571	SSR4	Translocon-associated protein subunit delta	0.50	18.999	ER<br>Extracellular region	Calcium binding	-
96.	P02786	TFRC	Transferrin receptor protein 1	0.62	84.871	PM<br>endosome	RNA binding	cellular iron ion homeostasis
97.	Q8NBS9	TXNDC5	Thioredoxin domain-containing protein 5	0.63	47.629	ER<br>Extracellular region	isomerase activity	apoptotic cell clearance
98.	O95573	ACSL3	Long-chain-fatty-acid--CoA ligase 3	2.00	80.420	Mitochondria<br>ER	long-chain fatty acid-CoA ligase activity	long-chain fatty acid import into cell
99.	P00966	ASS1	Argininosuccinate synthase	2.50	46.530	Cytosol<br>ER	ATP binding	arginine biosynthetic process
100.	Q07065	CKAP4	Cytoskeleton-associated protein 4	2.67	66.022	Cytokeleton<br>PM	RNA binding	post-translational protein modification
101.	P10606	COX5B	Cytochrome c oxidase subunit 5B, mitochondrial	2.00	13.696	Mitochondria	cytochrome-c oxidase activity	mitochondrial ATP synthesis coupled proton transport
102.	Q14697	GANAB	Neutral alpha-glucosidase AB	2.00	106.874	ER<br>Extracellular region	RNA binding	N-glycan processing
103.	P16403	HIST1H1C	Histone H1.2	2.40	21.365	Nucleus<br>Chromosome	RNA binding	chromosome condensation
104.	P16401	HIST1H1B	Histone H1.5	1.75	22.580	Nucleus<br>Chromosome	RNA binding	chromatin organization
105.	P04792	HSPB1	Heat shock protein beta-1	1.86	22.783	Cytoskeleton<br>Nucleus	RNA binding	anterograde axonal protein transport
106.	P02545	LMNA	Prelamin-A/C	1.80	74.139	Nucleus	identical protein binding	cellular protein localization

						Cytoskeleton		
107.	Q96AG4	LRRC59	Leucine-rich repeat-containing protein 59	2.33	34.930	Nucleus<br><br>ER	RNA binding	-
108.	P35579	MYH9	Myosin-9	2.00	226.532	Cytoskeleton<br><br>Cytosol	ADP binding	actin cytoskeleton reorganization
109.	P30101	PDIA3	Protein disulfide-isomerase A3	2.67	56.782	ER<br><br>Endosome	RNA binding	Protein folding
110.	P13667	PDIA4	Protein disulfide-isomerase A4	2.67	72.932	ER<br><br>Extracellular region	RNA binding	Protein folding
111.	Q15149	PLEC	Plectin	1.75	531.791	Cytoskeleton<br><br>Cytosol	RNA binding	hemidesmosome assembly
112.	P23284	PPIB	Peptidyl-prolyl cis-trans isomerase B	2.40	23.743	ER<br><br>Extracellular region	RNA binding	bone development
113.	Q13162	PRDX4	Peroxiredoxin-4	2.50	30.540	ER<br><br>cytosol	thioredoxin peroxidase activity	cell redox homeostasis
114.	P62424	RPL7A	60S ribosomal protein L7a	1.75	29.996	Cytosol<br><br>nucleus	RNA binding	Translation
115.	P04844	RPN2	Dolichyl-diphosphooligosaccharide--protein glycosyltransferase subunit 2	2.00	69.284	ER	Ribosome binding	cellular protein modification process
116.	P61247	RPS3A	40S ribosomal protein S3a	2.33	29.945	Nucleus<br><br>cytosol	RNA binding	Translation
117.	Q8NBS9	TXNDC5	Thioredoxin domain-containing protein 5	1.67	47.692	ER<br><br>Extracellular region	isomerase activity	apoptotic cell clearance
118.	P08670	VIM	Vimentin	1.95	53.652	Cytoskeleton<br><br>Nucleus	double-stranded RNA binding	positive regulation of translation
119.	P01891	HLA-A	HLA class I histocompati	0.56	40.909	ER	RNA binding	antigen processing and presentation of

			bility antigen, A-68 alpha chain			endosome		endogenous peptide antigen via MHC class I via ER pathway, TAP-independent
120.	P68032	ACTC1	Actin, alpha cardiac muscle 1	0.61	42.019	Cytoskeleton<br><br>Cytosol	ATP binding	actin filament organization
121.	P48047	ATP5PO	ATP synthase subunit O, mitochondrial	0.67	23.277	Mitochondria<br><br>Nucleus	ATPase binding	ATP5PO
122.	P27824	CANX	Calnexin	0.53	67.568	ER<br><br>Extracellular region	RNA binding	Protein folding
123.	P13987	CD59	CD59 glycoprotein	0.67	14.177	PM<br><br>Extracellular region	complement binding	cell surface receptor signaling pathway
124.	P40939	HADHA	Trifunctional enzyme subunit alpha, mitochondrial	0.67	83.000	Mitochondria	3-hydroxyacyl-CoA dehydrogenase activity	fatty acid beta-oxidation
125.	P58107	EPPK1	Epiplakin	0.40	555.658	Cytoskeleton<br><br>PM	RNA binding	intermediate filament bundle assembly
126.	P84090	ERH	Enhancer of rudimentary homolog	0.50	12.259	Methylosome	RNA binding	Cell cycle
127.	P20671	HIST1H2AD	Histone H2A type 1-D	0.63	14.107	Nucleus<br><br>ER	DNA binding	chromatin organization
128.	Q6FI13	HIST2H2AA3	Histone H2A type 2-A	0.63	14.095	Nucleus<br><br>ER	DNA binding	chromatin organization
129.	O75367	H2AFY	Core histone macro-H2A.1	0.60	39.617	Nucleus<br><br>ER	DNA binding	chromatin organization
130.	P33778	HIST1H2BB	Histone H2B type 1-B	0.48	13.950	Nucleus<br><br>cytosol	DNA binding	nucleosome assembly
131.	P62807	HIST1H2BC	Histone H2B type 1-C/E/F/G/I	0.54	13.906	Nucleus<br><br>cytosol	DNA binding	nucleosome assembly

132.	P69905	HBA1	Hemoglobin subunit alpha	0.53	15.258	Cytosol<br><br>Extracellular region	heme binding	Oxygen transport
133.	P46940	IQGAP1	Ras GTPase-activating-like protein IQGAP1	0.67	189.252	Nucleus<br><br>Cytoskeleton	GTPase activator activity	Signal transduction
134.	P55209	NAP1L1	Nucleosome assembly protein 1-like 1	0.67	45.374	Nucleus<br><br>Cytoplasm	RNA binding	DNA replication
135.	Q15366	PCBP2	Poly(rC)-binding protein 2	0.67	38.580	Nucleus<br><br>Extracellular region	RNA binding	mRNA metabolic process
136.	Q99623	PHB2	Prohibitin-2	0.67	33.296	Nucleus<br><br>Mitochondria	Amide binding	Protein stabilization
137.	P32119	PRDX2	Peroxiredoxin-2	0.67	21.892	Cytosol<br><br>Extracellular region	antioxidant activity	cell redox homeostasis
138.	P07737	PFN1	Profilin-1	0.50	15.045	Cytoskeleton<br><br>Cytosol	RNA binding	protein stabilization
139.	P20742	PZP	Pregnancy zone protein	0.57	163.863	Extracellular region	endopeptidase inhibitor activity	female pregnancy
140.	P26373	RPL13	60S ribosomal protein L13	0.63	24.261	Cytosol<br><br>ER	RNA binding	Translation
141.	P83731	RPL24	60S ribosomal protein L24	0.57	17.779	Cytosol<br><br>ER	RNA binding	Translation
142.	Q13501	SQSTM1	Sequestosome-1	0.50	47.687	Cytosol<br><br>Nucleus	SH2 domain binding	Aggrephagy

Table S2. Tame type proteins (CW).

Sr. No	Code	Gene name	Protein name	Ratio	Mol. masses kDa	Location	Molecular Function	Biological function
1.	P26447	S100A4	S100-A4	1.71	11.729	Extracellular region or secreted<br>Nucleus	RNA binding	positive regulation of I-kappaB kinase/NF-kappaB signaling
2.	P01891	HLA-A	1A68	1.80	40.909	ER<br>PM	RNA binding	regulation of immune response
3.	P18669	PGAM1	Phosphoglycerate mutase 1	2.00	28.804	Cytosol<br>ER	phosphoglycerate mutase activity	glycolytic process
4.	P32119	PRDX2	Peroxiredoxin-2	2.00	21.892	Cytoplasm<br>cytosol	antioxidant activity	cell redox homeostasis
5.	O43175	PHGDH	D-3-phosphoglycerate dehydrogenase	2.00	56.651	Cytosol<br>ER	phosphoglycerate dehydrogenase activity	Brain development
6.	P69905	HBA1	Hemoglobin subunit alpha	2.11	15.258	Cytosol<br>ER	Heme binding	Oxygen transport
7.	P60842	EIF4A1	Eukaryotic initiation factor 4A-I	2.50	46.154	Cytosol<br>ER	RNA binding	cytoplasmic translational initiation
8.	Q07955	SRSF1	Serine/arginine-rich splicing factor 1	3.50	27.745	Nucleus<br>Cytoplasm	RNA binding	alternative mRNA splicing, via spliceosome
9.	Q13162	PRDX4	Peroxiredoxin-4	0.29	30.540	ER<br>cytoplam	thioredoxin peroxidase activity	cell redox homeostasis
10.	P62424	RPL7A	60S ribosomal protein L7a	0.29	29.996	Cytosol<br>Nucleus	RNA binding	Translation
11.	P23284	PPIB	Peptidyl-prolyl cis-trans isomerase B	0.29	23.743	ER<br>Extracellular region	RNA binding	Bone development
12.	Q07065	CKAP4	Cytoskeleton-associated protein 4	0.30	66.022	Cytoskeleton	RNA binding	Post translation protein modification

						PM		
13.	Q96AG4	LRRC59	Leucine-rich repeat-containin g protein 59	0.33	34.930	Nucleus<br><br>ER	RNA binding	-
14.	P16401	HIST1H1B	Histone H1.5	0.36	22.580	Nucleus<br><br>Chromoso me	RNA binding	Chromatin organization
15.	Q8NBS9	TXNDC5	Thioredoxin domain-containing protein 5	0.38	47.629	ER<br><br>Extracellula r region	isomerase activity	apoptotic cell clearance
16.	P04792	HSPB1	Heat shock protein beta-1	0.39	22.783	Cytoskeleto n<br><br>Nucleus	RNA binding	anterograde axonal protein transport
17.	Q14697	GANAB	Neutral alpha-glucosidase AB	0.40	106.874	ER<br><br>Golgi apparatus	RNA binding	N-glycan processing
18.	P30101	PDIA3	Protein disulfide-isomerase A3	0.40	56.782	ER<br><br>Endosome	RNA binding	Protein folding
19.	P36578	RPL4	60S ribosomal protein L4	0.40	47.697	Cytosol<br><br>ER	RNA binding	Translation
20.	P35579	MYH9	Myosin-9	0.43	226.532	Cytoskeleto n<br><br>cytosol	RNA binding	actin cytoskeleton reorganization
21.	Q15084	PDIA6	Protein disulfide-isomerase A6	0.45	48.121	ER<br><br>PM	peptide disulfide oxidoreductase activity	Protein folding
22.	P00966	ASS1	Argininosucc inate synthase	0.46	46.530	Cytosol<br><br>ER	RNA binding	arginine biosynthetic process
23.	P31327	CPS1	Carbamoyl-phosphate synthase [ammonia], mitochondri al	0.49	164.939	Mitochondr ia<br><br>Nucleus	ATP binding	carbamoyl phosphate biosynthetic process
24.	O43852	CALU	Calumenin	0.50	37.107	ER<br><br>Golgi apparatus	calcium ion binding	cellular protein metabolic process

25.	P10606	COX5B	Cytochrome c oxidase subunit 5B, mitochondrial	0.50	13.696	Mitochondria	cytochrome-c oxidase activity	mitochondrial ATP synthesis coupled proton transport
26.	P05556	ITGB1	Integrin beta-1	0.50	88.415	Endosome<br><br>PM	Actin binding	B cell differentiation
27.	P20700	LMNB1	Lamin-B1	0.50	66.408	Nucleus<br><br>Cytoskeleton	structural molecule activity	interleukin-12-mediated signaling pathway
28.	P32322	PYCR1	Pyrroline-5-carboxylate reductase 1, mitochondrial	0.50	33.361	Mitochondria	identical protein binding	L-proline biosynthetic process
29.	O75340	PDCD6	Programmed cell death protein 6	0.50	21.868	Nucleus<br><br>endosome	Calcium ion binding	COPII vesicle coating
30.	Q15149	PLEC	Plectin	0.50	531.791	Cytoskeleton<br><br>Cytosol	RNA binding	hemidesmosome assembly
31.	P39023	RPL3	60S ribosomal protein L3	0.50	46.109	Nucleus<br><br>Cytosol	RNA binding	Translation
32.	P62851	RPS25	40S ribosomal protein S25	0.50	13.742	Cytosol<br><br>Extracellular region	RNA binding	Translation
33.	P61247	RPS3A	40S ribosomal protein S3a	0.50	29.945	Nucleus<br><br>ER	RNA binding	Translation
34.	P08670	VIM	Vimentin	0.53	53.652	Cytoskeleton<br><br>Nucleus	double-stranded RNA binding	positive regulation of translation
35.	P61006	RAB8A	Ras-related protein Rab-8A	0.56	23.668	Cytoskeleton<br><br>Endosome	GTP binding	Rab protein signal transduction
36.	P18124	RPL7	60S ribosomal protein L7	0.56	29.226	Cytosol<br><br>nucleus	DNA binding RNA binding	Translation
37.	P62280	RPS11	40S ribosomal protein S11	0.57	18.431	Cytosol	RNA binding	Translation

						Extracellular region		
38.	P11142	HSPA8	Heat shock cognate 71 kDa protein	0.58	70.898	Plasma membrane<br><br>Nucleus	ATP binding	ATP metabolic process
39.	P15311	EZR	Ezrin	0.60	69.413	Cytoskeleton<br><br>PM	ATPase binding	actin cytoskeleton reorganization
40.	Q99714	HSD17B10	3-hydroxyacyl-CoA dehydrogenase type-2	0.60	26.923	Mitochondria<br><br>PM	3-hydroxyacyl-CoA dehydrogenase activity	lipid metabolic process
41.	P55209	NAP1L1	Nucleosome assembly protein 1-like 1	0.60	45.374	Nucleus<br><br>Cytoplasm	RNA binding	DNA replication
42.	P08238	HSP90AB1	Heat shock protein HSP 90-beta	0.62	83.264	PM<br><br>Extracellular region	ATP binding	Protein folding
43.	P80723	BASP1	Brain acid soluble protein 1	0.63	22.693	PM<br><br>Extracellular region	protein domain specific binding	diaphragm development
44.	P02545	LMNA	Prelamin-A/C	0.63	74.139	Nucleus<br><br>cytoskeleton	identical protein binding	cellular protein localization
45.	P07237	P4HB	Protein disulfide-isomerase	0.63	57.116	PM<br><br>ER	RNA binding	Protein folding
46.	P09923	ALPI	Intestinal-type alkaline phosphatase	0.63	56.812	PM<br><br>Extracellular region	alkaline phosphatase activity	dephosphorylation
47.	P05186	ALPL	Alkaline phosphatase, tissue-nonspecific isozyme	0.63	57.305	PM<br><br>Extracellular region	alkaline phosphatase activity	skeletal system development
48.	P38646	HSPA9	Stress-70 protein, mitochondrial	0.65	73.680	Mitochondria<br><br>Nucleus	ATP binding	cellular response to heat

49.	P14618	PKM	Pyruvate kinase PKM	0.65	57.937	Mitochondrio<br><br>nucleus	ATP binding	ATP biosynthetic process
50.	P0DMV8	HSPA1A	Heat shock 70 kDa protein 1A	0.65	70.052	Cytoskeleton<br><br>Nucleus	ATP binding	ATP metabolic process
51.	P40926	MDH2	Malate dehydrogenase, mitochondrial	0.65	35.503	Mitochondria<br><br>Extracellular region	L-malate dehydrogenase activity	aerobic respiration
52.	P07355	ANXA2	Annexin A2	0.65	38.604	Extracellular region or secreted<br><br>Cytosol	RNA binding	Angiogenesis
53.	P49411	TUFM	Elongation factor Tu, mitochondrial	0.67	49.542	Mitochondria<br><br>Extracellular region	GTP binding	mitochondrial translational elongation
54.	P35268	RPL22	60S ribosomal protein L22	0.67	14.787	Cytosol<br><br>Extracellular region	RNA binding	Translation
55.	P04843	RPN1	Dolichyl-diphosphooligosaccharide--protein glycosyltransferase subunit 1	0.67	68.569	ER<br><br>Cytosol	RNA binding	cellular protein modification process
56.	P45880	VDAC2	Voltage-dependent anion-selective channel protein 2	0.67	31.567	Mitrochondria	voltage-gated anion channel activity	anion transport

Table S1. Mild type proteins (CY).

Sr. no	Code	Gene Name	Protein Name	Ratio	Mol.mass	Location	Molecular Function	Bio. Function
1.	P08133	ANAX6	Annexin A6	1.75	75.873	Cytoplasm Endosome	GTP binding	apoptotic signaling pathway
2.	P00533	EGFR	Epidermal growth factor receptor	2.33	134.277	E.R PM	ATP binding	activation of phospholipase A2 activity by calcium-mediated signaling
3.	P16403	HIST1H1C	Histone H1.2	1.71	21.365	Nucleus Chromosome	RNA binding	chromosome condensation
4.	P60842	EIF4A1	Eukaryotic initiation factor 4A-I	2.50	46.154	Cytosol Extracellular region	RNA binding	cytoplasmic translational initiation
5.	P13667	PDIA4	Protein disulfide-isomerase A4	2.00	72.932	ER Extracellular region	RNA binding	Protein folding
6.	P00558	PGK1	Phosphoglycerate kinase 1	3.00	44.615	Cytoplasm Cytosol	ADP binding	cellular response to hypoxia
7.	P62249	RPS16	40S ribosomal protein S16	1.67	16.445	Cytosol Extracellular region	RNA binding	translation
8.	Q07955	SRSF1	Serine/arginine-rich splicing factor 1	2.50	27.745	Nucleus Cytoplasm	DNA topoisomerase binding	alternative mRNA splicing, via spliceosome
9.	P27105	STOM	Erythrocyte band 7 integral membrane protein	2.00	31.731	Cytoskeleton PM	RNA polymerase binding	protein homooligomerization
10.	Q09666	AHNAK	Neuroblast differentiation-associated protein AHNAK	0.37	629.101	Nucleus Cytoskeleton	RNA binding	protein complex oligomerization

11.	P80723	BASP1	Brain acid soluble protein 1	0.63	22.693	PM<br><br>Cytoskeleton	protein domain specific binding	negative regulation of transcription, DNA-templated
12.	O43852	CALU	Calumenin	0.50	37.107	ER<br><br>Golgi apparatus	calcium ion binding	cellular protein metabolic process
13.	P61604	HSPE1	10 kDa heat shock protein, mitochondria	0.64	10.932	Mitochondria<br><br>Extracellular region	ATP binding	Protein folding
14.	P58107	EPPK1	Epiplakin	0.50	555.658	Cytoskeleton<br><br>PM	RNA binding	intermediate filament bundle assembly
15.	P38646	HSPA9	Stress-70 protein, mitochondrial	0.65	73.680	Nucleus<br><br>Mitochondria	ATP binding	Protein folding
16.	P16401	HIST1H1B	Histone H1.5	0.64	22.580	Nucleus	RNA binding	chromatin organization
17.	P33778	HIST1H2BB	Histone H2B type 1-B	0.51	13.950	Nucleus<br><br>Cytosol	DNA binding	nucleosome assembly
18.	P62807	HIST1H2BC	Histone H2B type 1-C/E/F/G/I	0.53	13.906	Nucleus<br><br>Cytosol	DNA binding	antibacterial humoral response
19.	Q99714	HSD17B10	3-hydroxyacyl-CoA dehydrogenase type-2	0.60	26.923	Mitochondria<br><br>PM	RNA binding	mitochondrial tRNA 3'-end processing
20.	P11279	LAMP1	Lysosome-associated membrane glycoprotein 1	0.40	44.882	Cytosol<br><br>Endosome	enzyme binding	Golgi to lysosome transport
21.	P55209	NAP1L1	Nucleosome assembly	0.40	45.374	Nucleus<br><br>cytoplasm	RNA binding	DNA binding

			protein 1-like 1					
22.	O75340	PDCD6	Programmed cell death protein 6	0.38	21.868	ER<br><br>Nucleus	calcium ion binding	COPII vesicle coating
23.	Q15084	PDIA6	Protein disulfide-isomerase A6	0.55	48.121	ER<br><br>PM	peptide disulfide oxidoreductase activity	Protein folding
24.	Q99623	PHB2	Prohibitin-2	0.62	33.296	Nucleus<br><br>Mitochondria	amide binding	mitochondrion organization
25.	P09923	ALP1	Intestinal-type alkaline phosphatase	0.63	56.812	PM<br><br>Extracellular region	alkaline phosphatase activity	dephosphorylation
26.	P30048	PRDX3	Thioredoxin-dependent peroxide reductase, mitochondrial	0.50	27.693	Endosome<br><br>Mitochondria	alkyl hydroperoxide reductase activity	cell redox homeostasis
27.	P07737	PFN1	Profilin-1	0.56	15.054	Cytoskeleton<br><br>cytosol	RNA binding	protein stabilization
28.	P61026	RAB10	Ras-related protein Rab-10	0.58	22.541	Cytoskeleton<br><br>ER	GDP binding	Rab protein signal transduction
29.	P51149	RAB7A	Ras-related protein Rab-7a	0.60	23.490	Endosome<br><br>Cytosol	GDP binding	Rab protein signal transduction
30.	P26373	RPL13	60S ribosomal protein L13	0.63	24.261	Cytosol<br><br>ER	RNA binding	Translation
31.	P46778	RPL21	60S ribosomal protein L21	0.60	18.565	ER<br><br>Cytosol	RNA binding	Translation
32.	P83731	RPL24	60S ribosomal protein L24	0.67	17.779	Cytosol<br><br>ER	RNA binding	Translation

33.	P36578	RPL4	60S ribosomal protein L4	0.40	47.697	Cytosol<br>ER	RNA binding	Translation
34.	P18124	RPL7	60S ribosomal protein L7	0.44	29.226	Cytosol<br>nucleus	DNA binding	Translation
35.	P62424	RPL7A	60S ribosomal protein L7a	0.50	29.996	Cytosol<br>Nucleus	RNA binding	Translation
36.	P52815	MRPL12	39S ribosomal protein L12, mitochondrial	0.67	21.348	Mitochondria	RNA binding	mitochondrial transcription
37.	P62280	RPS11	40S ribosomal protein S11	0.57	18.431	Cytosol<br>Extracellular region	RNA binding	translation
38.	P62244	RPS15A	40S ribosomal protein S15a	0.67	14.840	Cytosol<br>Extracellular region	RNA binding	Translation
39.	P51571	SSR4	Translocon-associated protein subunit delta	0.50	18.999	ER<br>Extracellular region	Calcium binding	-
40.	P02786	TFRC	Transferrin receptor protein 1	0.62	84.871	PM<br>endosome	RNA binding	cellular iron ion homeostasis
41.	Q8NBS9	TXNDC5	Thioredoxin domain-containing protein 5	0.63	47.629	ER<br>Extracellular region	isomerase activity	apoptotic cell clearance

Table S4. Wild Type Proteins (WY).

Sr. no	Code	Gene name	Protein name	Ratio	Mol. mass kDa	Location	Mol. function	Bio. Function
1.	O95573	ACSL3	Long-chain-fatty-acid--CoA ligase 3	2.00	80.420	Mitochondria<br>ER	long-chain fatty acid-CoA ligase activity	long-chain fatty acid import into cell
2.	P00966	ASS1	Argininosuccinate synthase	2.50	46.530	Cytosol<br>ER	ATP binding	arginine biosynthetic process
3.	Q07065	CKAP4	Cytoskeleton-associated protein 4	2.67	66.022	Cytokeleton<br>PM	RNA binding	post-translational protein modification
4.	P10606	COX5B	Cytochrome c oxidase subunit 5B, mitochondrial	2.00	13.696	Mitochondria	cytochrome-c oxidase activity	mitochondrial ATP synthesis coupled proton transport
5.	Q14697	GANAB	Neutral alpha-glucosidase AB	2.00	106.874	ER<br>Extracellular region	RNA binding	N-glycan processing
6.	P16403	HIST1H1C	Histone H1.2	2.40	21.365	Nucleus<br>Chromosome	RNA binding	chromosome condensation
7.	P16401	HIST1H1B	Histone H1.5	1.75	22.580	Nucleus<br>Chromosome	RNA binding	chromatin organization
8.	P04792	HSPB1	Heat shock protein beta-1	1.86	22.783	Cytoskeleton<br>Nucleus	RNA binding	anterograde axonal protein transport
9.	P02545	LMNA	Prelamin-A/C	1.80	74.139	Nucleus<br>Cytoskeleton	identical protein binding	cellular protein localization
10.	Q96AG4	LRRC59	Leucine-rich repeat-	2.33	34.930	Nucleus	RNA binding	-

			containing protein 59			ER		
11.	P35579	MYH9	Myosin-9	2.00	226.532	Cytoskeleton Cytosol	ADP binding	actin cytoskeleton reorganization
12.	P30101	PDIA3	Protein disulfide-isomerase A3	2.67	56.782	ER Endosome	RNA binding	Protein folding
13.	P13667	PDIA4	Protein disulfide-isomerase A4	2.67	72.932	ER Extracellular region	RNA binding	Protein folding
14.	Q15149	PLEC	Plectin	1.75	531.791	Cytoskeleton Cytosol	RNA binding	hemidesmosome assembly
15.	P23284	PPIB	Peptidyl-prolyl cis-trans isomerase B	2.40	23.743	ER Extracellular region	RNA binding	bone development
16.	Q13162	PRDX4	Peroxiredoxin-4	2.50	30.540	ER cytosol	thioredoxin peroxidase activity	cell redox homeostasis
17.	P62424	RPL7A	60S ribosomal protein L7a	1.75	29.996	Cytosol nucleus	RNA binding	translation
18.	P04844	RPN2	Dolichyl-diphosphooligosaccharide--protein glycosyltransferase subunit 2	2.00	69.284	ER	Ribosome binding	cellular protein modification process
19.	P61247	RPS3A	40S ribosomal protein S3a	2.33	29.945	Nucleus cytosol	RNA binding	translation
20.	Q8NBS9	TXNDC5	Thioredoxin domain-containing protein 5	1.67	47.692	ER Extracellular region	isomerase activity	apoptotic cell clearance
21.	P08670	VIM	Vimentin	1.95	53.652	Cytoskeleton Nucleus	double-stranded RNA binding	positive regulation of translation

22.	P01891	HLA-A	HLA class I histocompatibility antigen, A-68 alpha chain	0.56	40.909	ER<br><br>endosome	RNA binding	antigen processing and presentation of endogenous peptide antigen via MHC class I via ER pathway, TAP-independent
23.	P68032	ACTC1	Actin, alpha cardiac muscle 1	0.61	42.019	Cytoskeleton<br><br>Cytosol	ATP binding	actin filament organization
24.	P48047	ATP5PO	ATP synthase subunit O, mitochondrial	0.67	23.277	Mitochondria<br><br>Nucleus	ATPase binding	ATP5PO
25.	P27824	CANX	Calnexin	0.53	67.568	ER<br><br>Extracellular region	RNA binding	Protein folding
26.	P13987	CD59	CD59 glycoprotein	0.67	14.177	PM<br><br>Extracellular region	complement binding	cell surface receptor signaling pathway
27.	P40939	HADHA	Trifunctional enzyme subunit alpha, mitochondrial	0.67	83.000	Mitochondria	3-hydroxyacyl-CoA dehydrogenase activity	fatty acid beta-oxidation
28.	P58107	EPPK1	Epiplakin	0.40	555.658	Cytoskeleton<br><br>PM	RNA binding	intermediate filament bundle assembly
29.	P84090	ERH	Enhancer of rudimentary homolog	0.50	12.259	Methylosome	RNA binding	Cell cycle
30.	P20671	HIST1H2AD	Histone H2A type 1-D	0.63	14.107	Nucleus<br><br>ER	DNA binding	chromatin organization
31.	Q6FI13	HIST2H2AA3	Histone H2A type 2-A	0.63	14.095	Nucleus<br><br>ER	DNA binding	chromatin organization
32.	O75367	H2AFY	Core histone macro-H2A.1	0.60	39.617	Nucleus<br><br>ER	DNA binding	chromatin organization

33.	P33778	HIST1H2BB	Histone H2B type 1-B	0.48	13.950	Nucleus<br>cytosol	DNA binding	nucleosome assembly
34.	P62807	HIST1H2BC	Histone H2B type 1-C/E/F/G/I	0.54	13.906	Nucleus<br>cytosol	DNA binding	nucleosome assembly
35.	P69905	HBA1	Hemoglobin subunit alpha	0.53	15.258	Cytosol<br>Extracellular region	heme binding	Oxygen transport
36.	P46940	IQGAP1	Ras GTPase-activating-like protein IQGAP1	0.67	189.252	Nucleus<br>Cytoskeleton	GTPase activator activity	Signal transduction
37.	P55209	NAP1L1	Nucleosome assembly protein 1-like 1	0.67	45.374	Nucleus<br>Cytoplasm	RNA binding	DNA replication
38.	Q15366	PCBP2	Poly(rC)-binding protein 2	0.67	38.580	Nucleus<br>Extracellular region	RNA binding	mRNA metabolic process
39.	Q99623	PHB2	Prohibitin-2	0.67	33.296	Nucleus<br>Mitochondria	Amide binding	Protein stablization
40.	P32119	PRDX2	Peroxiredoxin-2	0.67	21.892	Cytosol<br>Extracellular region	antioxidant activity	cell redox homeostasis
41.	P07737	PFN1	Profilin-1	0.50	15.045	Cytoskeleton<br>Cytosol	RNA binding	protein stabilization
42.	P20742	PZP	Pregnancy zone protein	0.57	163.863	Extracellular region	endopeptidase inhibitor activity	female pregnancy
43.	P26373	RPL13	60S ribosomal protein L13	0.63	24.261	Cytosol<br>ER	RNA binding	translation
44.	P83731	RPL24	60S ribosomal protein L24	0.57	17.779	Cytosol<br>ER	RNA binding	Translation
45.	Q13501	SQSTM1	Sequestosome-1	0.50	47.687	Cytosol<br>Nucleus	SH2 domain binding	aggrephagy

Table S5. Common proteins in CW-CY.

Sr. No	Code	Gene name	Protein name	Ratio	Mol. masses kDa	Location	Molecular Function	Biological function
1.	P60842	EIF4A1	Eukaryotic initiation factor 4A-I	2.50	46.154	cytosol	RNA binding	cytoplasmic translational initiation
2.	Q07955	SRSF1	Serine/arginine-rich splicing factor 1	3.50	27.745	nucleus	RNA binding	alternative mRNA splicing, via spliceosome
3.	P36578	RPL4	60S ribosomal protein L4	0.40	47.697	Cytosol	RNA binding	translation
4.	Q15084	PDIA6	Protein disulfide-isomerase A6	0.45	48.121	ER	peptide disulfide oxidoreductase activity	Protein folding
5.	O43852	CALU	Calumenin	0.50	37.107	ER	calcium ion binding	cellular protein metabolic process
6.	O75340	PDCD6	Programmed cell death protein 6	0.50	21.868	endosome	Calcium ion binding	COPII vesicle coating
7.	P18124	RPL7	60S ribosomal protein L7	0.56	29.226	cytosol	DNA binding RNA binding	Translation
8.	P62280	RPS11	40S ribosomal protein S11	0.57	18.431	cytosol	RNA binding	Translation
9.	Q99714	HSD17B10	3-hydroxyacyl-CoA dehydrogenase type-2	0.60	26.923	mitochondria	3-hydroxyacyl-CoA dehydrogenase activity	lipid metabolic process
10.	P80723	BASP1	Brain acid soluble protein 1	0.63	22.693	PM	protein domain specific binding	diaphragm development
11.	P09923	ALPI	Intestinal-type alkaline phosphatase	0.63	56.812	PM	alkaline phosphatase activity	Dephosphorylation
12.	P38646	HSPA9	Stress-70 protein, mitochondrial	0.65	73.680	nucleus	ATP binding	cellular response to heat

Table S6. Common proteins in CW-WY.

Sr. No	Code	Gene Name	Protein Name	Ratio	Mol.mass	Location	Mol. function	Bio. Function
1.	P16403	HIST1H1C	Histone H1.2	1.71	21.365	nucleus	RNA binding	chromosome condensation
2.	P13667	PDIA4	Protein disulfide-isomerase A4	2.00	72.932	ER	RNA binding	Protein folding
3.	P58107	EPPK1	Epiplakin	0.50	555.658	cytoskeleton	RNA binding	intermediate filament bundle assembly
4.	P33778	HIST1H2BB	Histone H2B type 1-B	0.51	13.950	nucleus	DNA binding	nucleosome assembly
5.	P62807	HIST1H2BC	Histone H2B type 1-C/E/F/G/I	0.53	13.906	nucleus	DNA binding	antibacterial humoral response
6.	Q99623	PHB2	Prohibitin-2	0.62	33.296	nucleus	amide binding	mitochondrion organization
7.	P07737	PFN1	Profilin-1	0.56	15.054	Cytoskeleton	RNA binding	protein stabilization
8.	P26373	RPL13	60S ribosomal protein L13	0.63	24.261	cytosol	RNA binding	Translation
9.	P83731	RPL24	60S ribosomal protein L24	0.67	17.779	Cytosol	RNA binding	Translation

Table S7. Common proteins in CY – WY.

Sr.<br><br>No	Code	Gene name	Protein name	Ratio	Mol.<br><br>masses kDa	Location	Molecular Function	Biological function
1.	P01891	HLA-A	1A68	1.80	40.909	ER	RNA binding	regulation of immune response
2.	P32119	PRDX2	Peroxiredoxin-2	2.00	21.892	cytoplasm	antioxidant activity	cell redox homeostasis
3.	P69905	HBA1	Hemoglobin subunit alpha	2.11	15.258	cytosol	Heme binding	Oxygen transport
4.	Q13162	PRDX4	Peroxiredoxin-4	0.29	30.540	ER	thioredoxin peroxidase activity	cell redox homeostasis
5.	P23284	PPIB	Peptidyl-prolyl cis-trans isomerase B	0.29	23.743	ER	RNA binding	Bone development
6.	Q07065	CKAP4	Cytoskeleton-associated protein 4	0.30	66.022	cytoskeleton	RNA binding	Post translation protein modification
7.	Q96AG4	LRRC59	Leucine-rich repeat-containing protein 59	0.33	34.930	nucleus	RNA binding	-
8.	P04792	HSPB1	Heat shock protein beta-1	0.39	22.783	cytoskeleton	RNA binding	anterograde axonal protein transport
9.	Q14697	GANAB	Neutral alpha-glucosidase AB	0.40	106.874	ER	RNA binding	N-glycan processing
10.	P30101	PDIA3	Protein disulfide-isomerase A3	0.40	56.782	ER	RNA binding	Protein folding
11.	P35579	MYH9	Myosin-9	0.43	226.532	cytoskeleton	RNA binding	actin cytoskeleton reorganization
12.	P00966	ASS1	Argininosuccinate synthase	0.46	46.530	cytosol	RNA binding	arginine biosynthetic process
13.	P10606	COX5B	Cytochrome c oxidase subunit 5B, mitochondrial	0.50	13.696	Mitochondria	cytochrome-c oxidase activity	mitochondrial ATP synthesis coupled proton transport

14.	Q15149	PLEC	Plectin	0.50	531.791	cytoskeleton	RNA binding	hemidesmosome assembly
15.	P61247	RPS3A	40S ribosomal protein S3a	0.50	29.945	nucleus	RNA binding	Translation
16.	P08670	VIM	Vimentin	0.53	53.652	cytoskeleton	double-stranded RNA binding	positive regulation of translation
17.	P02545	LMNA	Prelamin-A/C	0.63	74.139	Nucleus	identical protein binding	cellular protein localization

Table S8. Common proteins in CWY.

Sr. No	Code	Gene name	Protein name	Ratio	Mol. masses kDa	Location	Molecular Function	Biological function
1.	P62424	RPL7A	60S ribosomal protein L7a	0.29	29.996	cytosol	RNA binding	translation
2.	P16401	HIST1H1B	Histone H1.5	0.36	22.580	nucleus	RNA binding	Chromatin organization
3.	Q8NBS9	TXNDC5	Thioredoxin domain-containing protein 5	0.38	47.629	ER	isomerase activity	apoptotic cell clearance
4.	P55209	NAP1L1	Nucleosome assembly protein 1-like 1	0.60	45.374	nucleus	RNA binding	DNA replication

Table S9. Unique Proteins in CW.

Sr. No	Code	Gene name	Protein name	Ratio	Mol. masses kDa	Location	Molecular Function	Biological function
1.	P26447	S100A4	S100-A4	1.71	11.729	Extracellular region	RNA binding	positive regulation of I-kappaB kinase/NF-kappaB signaling
2.	P18669	PGAM1	Phosphoglycerate mutase 1	2.00	28.804	cytosol	phosphoglycerate mutase activity	glycolytic process
3.	O43175	PHGDH	D-3-phosphoglycerate dehydrogenase	2.00	56.651	cytosol	phosphoglycerate dehydrogenase activity	Brain development
4.	P31327	CPS1	Carbamoyl-phosphate synthase [ammonia], mitochondrial	0.49	164.939	mitochondria	ATP binding	carbamoyl phosphate biosynthetic process
5.	P05556	ITGB1	Integrin beta-1	0.50	88.415	endosome	Actin binding	B cell differentiation
6.	P20700	LMNB1	Lamin-B1	0.50	66.408	nucleus	structural molecule activity	interleukin-12-mediated signaling pathway
7.	P32322	PYCR1	Pyrroline-5-carboxylate reductase 1, mitochondrial	0.50	33.361	mitochondria	identical protein binding	L-proline biosynthetic process
8.	P39023	RPL3	60S ribosomal protein L3	0.50	46.109	nucleus	RNA binding	Translation
9.	P62851	RPS25	40S ribosomal protein S25	0.50	13.742	cytosol	RNA binding	Translation
10.	P61006	RAB8A	Ras-related protein Rab-8A	0.56	23.668	endosome	GTP binding	Rab protein signal transduction
11.	P11142	HSPA8	Heat shock cognate 71 kDa protein	0.58	70.898	Plasma membrane	ATP binding	ATP metabolic process

12.	P15311	EZR	Ezrin	0.60	69.413	cytoskeleton	ATPase binding	actin cytoskeleton reorganization
13.	P08238	HSP90AB1	Heat shock protein HSP 90-beta	0.62	83.264	PM	ATP binding	Protein folding
14.	P07237	P4HB	Protein disulfide-isomerase	0.63	57.116	ER	RNA binding	Protein folding
15.	P05186	ALPL	Alkaline phosphatase, tissue-nonspecific isozyme	0.63	57.305	PM	alkaline phosphatase activity	skeletal system development
16.	P14618	PKM	Pyruvate kinase PKM	0.65	57.937	nucleus	ATP binding	ATP biosynthetic process
17.	P0DMV8	HSPA1A	Heat shock 70 kDa protein 1A	0.65	70.052	cytoskeleton	ATP binding	ATP metabolic process
18.	P40926	MDH2	Malate dehydrogenase, mitochondrial	0.65	35.503	mitochondria	L-malate dehydrogenase activity	aerobic respiration
19.	P07355	ANXA2	Annexin A2	0.65	38.604	Extracellular region or secreted	RNA binding	Angiogenesis
20.	P49411	TUFM	Elongation factor Tu, mitochondrial	0.67	49.542	mitochondria	GTP binding	mitochondrial translational elongation
21.	P35268	RPL22	60S ribosomal protein L22	0.67	14.787	cytosol	RNA binding	Translation
22.	P04843	RPN1	Dolichyl-diphosphooligosaccharide--protein glycosyltransferase subunit 1	0.67	68.569	ER	RNA binding	cellular protein modification process
23.	P45880	VDAC2	Voltage-dependent anion-selective channel protein 2	0.67	31.567	Mitrochondria	voltage-gated anion channel activity	anion transport

Table S2. Unique proteins in CY.

Sr. no	Code	Gene Name	Protein Name	Ratio	Mol.mass	Location	Mol. Function	Bio. Function
1.	P08133	ANAX6	Annexin A6	1.75	75.873	cytoplasm	GTP binding	apoptotic signaling pathway
2.	P00558	PGK1	Phosphoglycerate kinase 1	3.00	44.615	cytoplasm	ADP binding	cellular response to hypoxia
3.	P62249	RPS16	40S ribosomal protein S16	1.67	16.445	cytosol	RNA binding	translation
4.	P27105	STOM	Erythrocyte band 7 integral membrane protein	2.00	31.731	cytoskeleton	RNA polymerase binding	protein homooligomerization
5.	Q09666	AHNAK	Neuroblast differentiation-associated protein AHNAK	0.37	629.101	nucleus	RNA binding	protein complex oligomerization
6.	P61604	HSPE1	10 kDa heat shock protein, mitochondria	0.64	10.932	Mitochondria	ATP binding	Protein folding
7.	P11279	LAMP1	Lysosome-associated membrane glycoprotein 1	0.40	44.882	endosome	enzyme binding	Golgi to lysosome transport
8.	P30048	PRDX3	Thioredoxin-dependent peroxide reductase, mitochondrial	0.50	27.693	endosome	alkyl hydroperoxide reductase activity	cell redox homeostasis
9.	P61026	RAB10	Ras-related protein Rab-10	0.58	22.541	Cytoskeleton	GDP binding	Rab protein signal transduction
10.	P51149	RAB7A	Ras-related protein Rab-7a	0.60	23.490	endosome	GDP binding	Rab protein signal transduction

11.	P46778	RPL21	60S ribosomal protein L21	0.60	18.565	ER	RNA binding	Translation
12.	P52815	MRPL12	39S ribosomal protein L12, mitochondrial	0.67	21.348	Mitochondria	RNA binding	mitochondrial transcription
13.	P62244	RPS15A	40S ribosomal protein S15a	0.67	14.840	cytosol	RNA binding	Translation
14.	P51571	SSR4	Translocon-associated protein subunit delta	0.50	18.999	ER	Calcium binding	-
15.	P02786	TFRC	Transferrin receptor protein 1	0.62	84.871	PM	RNA binding	cellular iron ion homeostasis
16.	P00533	EGFR	Epidermal growth factor receptor	2.33	134.277	PM<br>ER	ATP binding	activation of phospholipase A2 activity by calcium-mediated signaling

Table S3. Unique proteins in WY.

**Sr. No**	**Code**	**Gene name**	**Protein name**	**Ratio**	**Mol. mass kDa**	**Location**	**Mol. function**	**Bio. Function**
1.	O95573	ACSL3	Long-chain-fatty-acid--CoA ligase 3	2.00	80.420	Mitochondria	long-chain fatty acid-CoA ligase activity	long-chain fatty acid import into cell
2.	P04844	RPN2	Dolichyl-diphosphooligosaccharide--protein glycosyltransferase subunit 2	2.00	69.284	ER	Ribosome binding	cellular protein modification process

3.	P68032	ACTC1	Actin, alpha cardiac muscle 1	0.61	42.019	Cytoskeleton	ATP binding	actin filament organization
4.	P48047	ATP5PO	ATP synthase subunit O, mitochondrial	0.67	23.277	Mitochondria	ATPase binding	ATP5PO
5.	P27824	CANX	Calnexin	0.53	67.568	ER	RNA binding	Protein folding
6.	P13987	CD59	CD59 glycoprotein	0.67	14.177	PM	complement binding	cell surface receptor signaling pathway
7.	P40939	HADHA	Trifunctional enzyme subunit alpha, mitochondrial	0.67	83.000	Mitochondria	3-hydroxyacyl-CoA dehydrogenase activity	fatty acid beta-oxidation
8.	P84090	ERH	Enhancer of rudimentary homolog	0.50	12.259	methylosome	RNA binding	Cell cycle
9.	P20671	HIST1H2AD	Histone H2A type 1-D	0.63	14.107	Nucleus	DNA binding	chromatin organization
10.	Q6FI13	HIST2H2AA3	Histone H2A type 2-A	0.63	14.095	Nucleus	DNA binding	chromatin organization
11.	O75367	H2AFY	Core histone macro-H2A.1	0.60	39.617	Nucleus	DNA binding	chromatin organization
12.	P46940	IQGAP1	Ras GTPase-activating-like protein IQGAP1	0.67	189.252	Nucleus	GTPase activator activity	Signal transduction
13.	Q15366	PCBP2	Poly(rC)-binding protein 2	0.67	38.580	Nucleus	RNA binding	mRNA metabolic process
14.	P20742	PZP	Pregnancy zone protein	0.57	163.863	Extracellular region	endopeptidase inhibitor activity	female pregnancy
15.	Q13501	SQSTM1	Sequestosome-1	0.50	47.687	Cytosol	SH2 domain binding	Aggrephagy
